# Recent Advances in Catalytic Conversion of Bioethanol to 1,3‐Butadiene: Reaction Mechanism, Catalyst Design, and Process Scalability

**DOI:** 10.1002/cssc.202501926

**Published:** 2025-11-26

**Authors:** Abhishek R. Varma, Md Ziyaur Rahman, Siddharth Gadkari, Atthasit Tawai, Malinee Sriariyanun, Ao Xia, Vinod Kumar, Sunil K. Maity

**Affiliations:** ^1^ Department of Chemical Engineering Indian Institute of Technology Hyderabad Sangareddy India; ^2^ Advanced Centre for Energetic Material (ACEM) Defence Research and Development Organisation (DRDO) Nashik India; ^3^ School of Chemistry and Chemical Engineering University of Surrey Guildford UK; ^4^ The Sirindhorn International Thai‐German Graduate School of Engineering King Mongkut's University of Technology North Bangkok Bangkok Thailand; ^5^ Institute of Engineering Thermophysics School of Energy and Power Engineering Chongqing University Chongqing China; ^6^ Magan Centre for Applied Mycology Faculty of Engineering and Applied Sciences Cranfield University Cranfield UK; ^7^ Centre for Sustainable Rural Development Indian Institute of Technology Roorkee Roorkee India

**Keywords:** 1,3‐butadiene, ethanol, heterogeneous catalyst, reaction mechanism, thermodynamic analysis

## Abstract

1,3‐Butadiene (BD), a symmetric C_4_ diene, is a primary precursor for numerous synthetic rubbers and is sourced largely from the naphtha cracking process. Sustainable BD production from renewable biomass is indispensable for preserving the environment through the circular economy. Ethanol‐to‐BD (ETB) has particularly witnessed a resurgence in recent years, following two different routes: one‐step conversion and two‐step process via acetaldehyde. The present review article critically examines the current state‐of‐the‐art research progress of the ETB processes, in terms of historical perspective, reaction mechanism, kinetics, thermodynamics, multifunctional heterogeneous catalysts, reaction parameters, and economic‐environmental impact analysis. The ETB processes encompass a complex sequence of reactions on different catalytic sites, including dehydrogenation, carbon–carbon coupling, and dehydration. However, the catalyst with the proper balance between acidic, basic, redox, and metal functionalities (e.g., metal/metal oxide‐modified MgO–SiO_2_ and Zn–Zr mixed oxide), which are uniformly distributed and cooperative, remains a critical challenge in these processes. Despite notable advancements in understanding molecular mechanisms, the design of catalysts for high BD selectivity and process scalability remains the key obstacle to commercial success. The comprehensive summary of ETB process developments provides a foundation for researchers and industry practitioners to advance research and optimize the critical parameters for sustainable BD production.

## Graphical abstract

Catalytic conversion of ethanol to 1,3‐butadiene (BD) can proceed via one‐step and two‐step routes. In the one‐step process, pure and metal‐modified MgO–SiO_2_ catalysts enable direct ethanol transformation to BD, while ZrO_2_‐based catalysts drive the two‐step pathway. Optimal BD selectivity requires a finely tuned balance of acidic, basic, and metallic sites to regulate reaction intermediates and minimize byproduct formation.

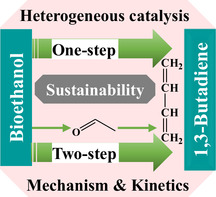

## 1 Introduction

1,3‐Butadiene (BD) is a conjugated and symmetric C_4_ diene (CH_2_=CH–CH = CH_2_). This diolefin is a vital building block with vast applications in the rubber and polymer industries. BD was first recognized as a feedstock for synthetic rubber in the 1910s [1]. The primary BD‐derived rubbers include styrene‐butadiene (SBR), acrylonitrile‐butadiene (ABR), nitrile (NR), and polychloroprene (neoprene) [2]. BD also finds applications in the synthesis of elastomers, plastics, and resins. The BD demand has increased rapidly in recent decades due to the fast‐growing downstream market, particularly in the Asia‐Pacific region [3]. The BD market is expected to expand from an estimated 14.11 million metric tons (MT) in 2024 to nearly 16.94 million MT by 2029 at a healthy compound annual growth rate of 3.71% [3]. However, over 95% of BD is cogenerated during steam naphtha cracking for ethylene production [4]. Nevertheless, the rising use of lighter hydrocarbons, such as shale gas, as preferred raw materials for ethylene manufacturing triggers a short supply of BD, resulting in soaring prices [5]. Moreover, over‐reliance on nonrenewable petroleum poses significant environmental challenges [6]. Therefore, sustainable BD production from renewable biomass bears incredible significance in meeting escalating demand for commodities and contributes to carbon neutrality.

**FIGURE 1:**
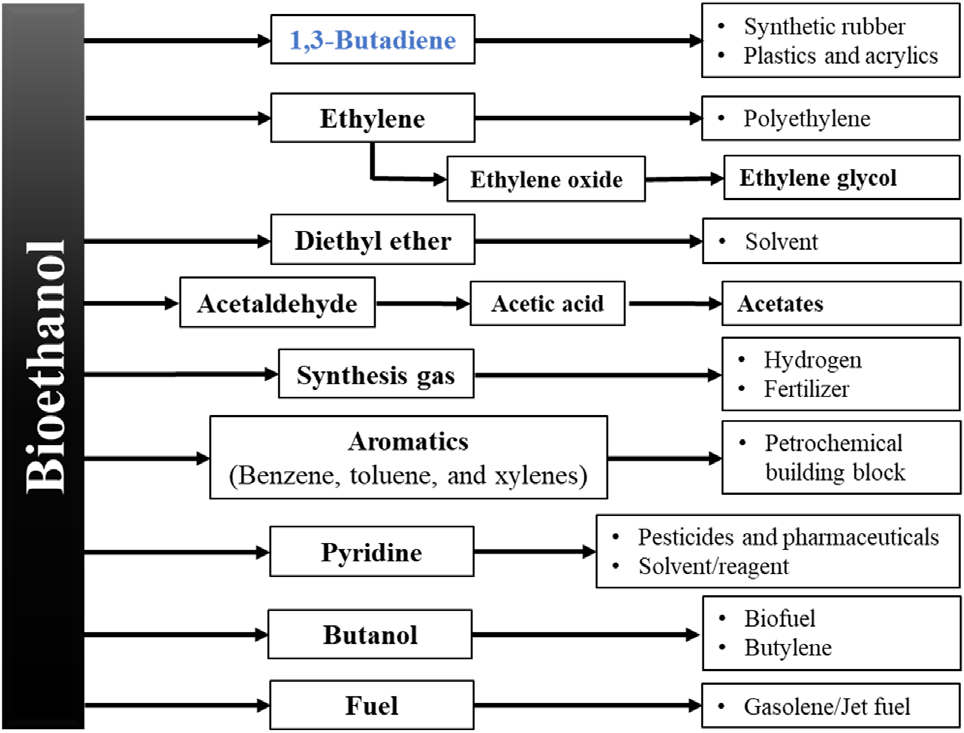
Derivative potential of bioethanol.

Biomass is an inexhaustible resource formed by the capture of atmospheric CO_2_ and holds excellent potential to provide chemicals, fuels, and energy in an integrated biorefinery. In 2024, the US Department of Energy listed the potential bio‐based molecules for sourcing renewable chemicals, known as platform chemicals [7]. The platform chemicals are small molecules and can readily be transformed into more complex products. Their publication triggered a paradigm shift in resource utilization, as evidenced by an expanding bioproduct market. Bioethanol is a vital platform chemical with vast derivative potential, including BD, and holds tremendous significance as a biofuel (Figure 1). The success of bioethanol is attributed to its high level of technological readiness. Bioethanol is produced by fermentation of C_6_ and C_5_ sugars using traditional or genetically modified yeasts or bacteria [8]. The carbohydrates are sourced from sugar, maize, starch, and cellulosic biomass. However, the high costs of cellulosic bioethanol remain a challenge for commercial applications due to the expensive pretreatment and hydrolysis steps [9].

**SCHEME 1:**
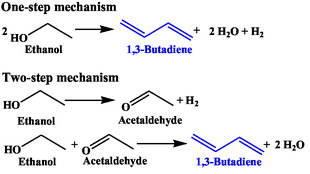
Overall reactions for one‐step and two‐step mechanisms.

BD production from ethanol was first reported by Russian scientist Ipatiev Lebedev in the early 1900s, following the discovery of BD conversion into rubber‐like polymers (Scheme 1). However, early studies suffered from a meagre 1.5% BD yield [10]. Thereafter, Lebedev's group made significant advancements in designing multifunctional catalysts with undisclosed composition, achieving a high BD yield with 70% selectivity under atmospheric pressure at 623 K [11]. Around the same time, Interessen‐Gemeinschaft Farbenindustrie AG (IG Farben) tested cobalt or chromium‐promoted magnesia catalysts and reported 60% BD yield [12]. A similar catalyst containing 59% magnesia, 2% chromic acid, and 39% silica gel was also examined, and 38% BD yield per pass with 56% selectivity was achieved at 673–698 K [12]. This process is referred to as the one‐step method or Russian process. A two‐step approach, popularly known as the Ostromislensky or American process, was developed during the Second World War in the US (Scheme 1) [13]. The process was jointly operated by Union Carbide, Carbon Chemicals Corporation, and Koppers Company. It comprised ethanol dehydrogenation, followed by the reaction of acetaldehyde with ethanol over tantala‐silica catalyst. The yield of acetaldehyde from ethanol dehydrogenation was 92%, with an overall BD yield of 60%–64% [14]. The higher BD yield with better purity was proclaimed for the two‐step process. However, the one‐step process was claimed to be simple with lower production costs due to fewer reactors and separating equipment.

Despite early discovery, commercial operation of the ethanol‐to‐BD (ETB) process was abandoned after the 1960s due to the emergence of more efficient and low‐cost petrochemical technologies. Currently, very few ETB plants are operational worldwide, mostly favored by regional economics. Scientific interest also waned, as evidenced by the limited published articles between 1960 and 2000. However, recent times have witnessed a resurgence in the ETB process due to the unsustainability of petroleum, soaring BD prices, and supply‐demand imbalance caused by dwindling naphtha‐based ethylene production, highlighting the potential for bio‐based BD manufacturing to meet global demand. The growing public concern about greenhouse gas (GHG) emissions further led to a net‐zero carbon emission policy framework by 2050, fueling BD production from bioethanol [15]. The sustainability analysis revealed a strong relationship between the price of BD and bioethanol. The BD production is economical in Brazil due to favorable bioethanol price [16, 17]. Economic analysis of the one‐step process revealed that an increase in BD selectivity (+11%) could reduce the ethanol consumption per ton of BD, CO_2_ emission (10%–14%), water consumption (18%), and cumulative energy demand (12%–14%) [16]. The ETB process was also cost‐competitive at current BD prices (0.5–1.23 USD/kg) when carbon credit was taken into account [16]. The Bio‐Butterfly project was the first ETB commercialization initiative by France, which was launched in 2012 by Michelin, IFP Energies Nouvelles, and its subsidiary Axens [18]. An industrial‐scale demonstration was announced recently at the Michelin site in Bassens [18].

To the best of our knowledge, dedicated review articles on the ETB processes are limited [13, 19, 20, 21]. The present article thus attempts to provide comprehensive and up‐to‐date research and technological status on green approaches for catalytic bioethanol conversion into BD. The review specifically outlines reaction mechanisms with their thermodynamic insights, explores kinetics, and discusses multifunctional catalysts. The review precisely focuses on the fundamental understanding of reaction mechanisms and the impact of various active sites on catalytic activity, product yield, and selectivity. The article finally includes concluding remarks, future outlook, and opportunities with an argument for an integrated biorefinery to broaden the spectrum of bio‐based products.

## 2 Reaction Mechanism

The BD was known to form in small quantities when ethanol was passed over aluminum powder and in fair amounts when an ethanol–acetaldehyde mixture was flowed over alumina/clay or ethanol alone was flowed over catalysts with dehydrogenation‐dehydration sites [22]. However, low BD yield and excessive coproducts were the major challenges at that time. Knowledge of the reaction mechanism is thus critical for improving BD yield, depending on the nature of the catalysts. It also facilitates logical catalyst formulation, kinetic modeling, and reactor design. The modern physicochemical methods and density functional theory (DFT) have further renewed interest in elucidating an accurate mechanism. This section examines proposed mechanisms, most of which, however, lack experimental evidence.

### 2.1 Lebedev Process

**SCHEME 2:**
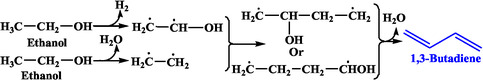
Lebedev mechanism.

**SCHEME 3:**
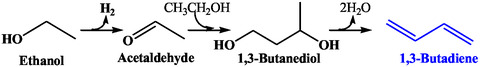
Egloff and Hulla's mechanism.

The inventor of the one‐step process, Lebedev, proposed BD formation through divalent radicals, formed by ethanol dehydrogenation and dehydration reactions (Scheme 2) [22]. These two‐carbon radicals then combine to form four‐carbon radicals, which dehydrate to BD [22]. This mechanism was inferred from Lebedev's own experiments, where a gradual increase in dehydration sites (with a simultaneous decrease in dehydrogenating centers) resulted in a rapid rise in BD yield with a corresponding decline in acetaldehyde. However, BD yield was reduced for a further increase in the dehydrating component, and the selectivity gradually moved toward ethylene. The proposed mechanism is thus expected to produce greater amounts of ethylene and butylene than observed, with many disagreements. In later years, based on multiplet theory, Balandin concluded that energy constraints would prevent the formation of ethanol radicals, and an alternate mechanism was proposed [23]. Balandin's mechanism involves ethanol dehydrogenation and acetaldehyde condensation with ethanol to 1,3‐butanediol (1,3‐BDO), followed by 1,3‐BDO dehydration to BD (Scheme 3) [23]. Egloff and Hulla extended Balandin's mechanism based on the dehydration component of the catalyst to include diverse intermediates, such as (1,3 or 1,4 or 2,3) butanediols, butenols (1‐buten‐3 (or 4)‐ol, 2‐buten‐1‐ol), and (1,3 or 1,4 or 2,3) epoxybutanes [22]. They further listed the multiple possible mechanisms using different combinations of these intermediates.

Another mechanism was proposed based on the synergistic action of dehydration and dehydrogenation, where ethanol was dehydrated to ethylene, followed by its dehydrogenation to acetylene [22]. BD was formed by the interaction of the unsaturated hydrocarbons. However, this mechanism is valid only under particular reaction conditions, especially in the presence of strong dehydrogenation components.

### 2.2 Ostromislensky Process

**SCHEME 4:**
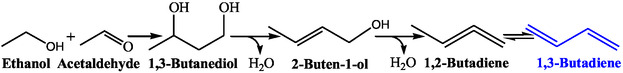
Ostromislensky mechanism.

The Ostromislensky process starts with the reaction between ethanol and acetaldehyde. Ostromislensky proposed that reaction was ensured through ethyl hydroxyethyl ether intermediate, formed by the ethanol–acetaldehyde interaction [22]. This intermediate was subsequently converted to 1,3‐BDO, which was dehydrated to BD via 2‐buten‐1‐ol (2B1OL). However, Ostromislensky latter reported that ethyl hydroxyethyl ether played no role in the BD formation as it readily decomposed into ethanol and acetaldehyde under reaction temperatures (Scheme 4). Although 1,3‐BDO was not observed, the Ostromislensky mechanism was supported by the formation of 2B1OL and 1,2‐butadiene, which could isomerize to BD [22]. They further studied the reaction using iso‐propanol to identify the exact carbon atoms involved in C–C coupling [24]. Their experiment demonstrated the formation of piperylene, rather than isoprene, indicating the involvement of the methyl group carbon of ethanol and the carbonyl carbon of acetaldehyde in C–C bond formation. Further investigation replaced acetaldehyde with paraldehyde or 1,2‐ethanediol, as they break down to acetaldehyde at high temperatures [22]. Therefore, the mechanism via 1,3‐ or 1,4‐butanediol cannot be ruled out in the latter case. Another study suggested acetylene as a probable intermediate for BD formation [22].

**SCHEME 5:**
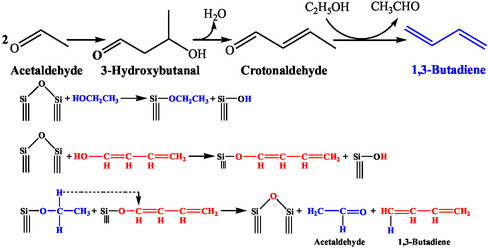
Quattlebaum mechanism via crotonaldehyde.

In later years, Quattlebaum et al. investigated various reactions to confirm the exact C–C coupling reaction responsible for BD formation [24]. They concluded that the mechanism ensued through crotonaldehyde, which was formed via dehydration of the acetaldehyde aldol condensation product, 3‐hydroxybutanal (Scheme 5). This mechanism was supported by the higher BD yield when crotonaldehyde was passed with ethanol over an alumina catalyst than from an acetaldehyde–ethanol mixture [24]. Crotonaldehyde was subsequently deoxygenated to BD by ethanol, which itself oxidized to acetaldehyde. The thermodynamic calculation also demonstrates that crotonaldehyde reduction by ethanol is more favorable than crotonaldehyde deoxygenation by molecular hydrogen [25]. While these facts support crotonaldehyde as the principal intermediate, it does not exclude the possibility of the reduction of 3‐hydroxybutanal to 1,3‐BDO, followed by its dehydration to BD [24]. Therefore, they extended the investigation using pure 1,3‐BDO. The dehydration of pure 1,3‐BDO showed a higher propylene/BD ratio compared to that of acetaldehyde‐ethanol, thereby ruling out the possibility of 1,3‐BDO intermediate [24]. Quattlebaum et al. further depicted the molecular deoxygenation mechanism over activated silica gel catalysts with ≡Si–O–Si≡ and ≡Si–OH surface species (Scheme 5) [24]. The reactive bridged siloxane was proposed to anchor the oxygen atom of ethanol and the enol of crotonaldehyde, followed by an intermolecular hydride shift from ethanol to crotonaldehyde enol to form BD. However, oxide promoters (e.g., tantalum, zirconium, or niobium oxides) perform the aldol condensation.

**SCHEME 6:**
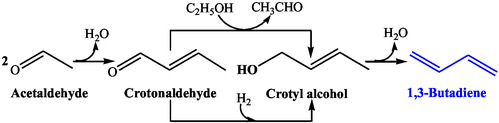
Natta and Rigamonti mechanism.

Natta and Rigamonti proposed that crotonaldehyde was formed directly from acetaldehyde [26]. However, they introduced an intermediate reaction step in the Quattlebaum mechanism, where crotonaldehyde was first reduced to crotyl alcohol via hydrogen transfer from ethanol or by molecular hydrogen, followed by its dehydration to BD (Scheme 6). However, the crotonaldehyde reduction by molecular hydrogen is less likely, as the reduction by ethanol is thermodynamically more favorable [25].

To explore the mechanistic insight, Niiyama et al. studied the reaction using the intermediates involved in various mechanisms over SiO_2_–MgO acid–base catalysts [27]. However, BD was not observed from pure 1,3‐BDO, ruling out the possibility of 1,3‐BDO intermediate. The reaction of acetaldehyde with isopropanol exhibited BD (and acetone) formation instead of isoprene or pentadiene, thereby eliminating the possibility of crotyl alcohol intermediate. This study concluded that the reaction followed the Quattlebaum et al. mechanism (Scheme 5). Their study further showed that adding acetaldehyde significantly sped up the reaction, demonstrating ethanol dehydrogenation as the rate‐limiting step.

**SCHEME 7:**
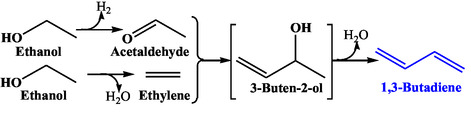
Prins‐condensation mechanism.

Kitayama et al. investigated ETB reaction using sepiolite and Mn‐sepiolite [28]. While sepiolite was selective to ethylene, Mn‐sepiolite was effective in BD formation. Gruver et al. further extended the study using aluminated sepiolite and Ag‐exchanged aluminated sepiolite [29]. BD yield was low over aluminated sepiolite, with diethyl ether (DEE) and ethylene being the dominating products, while BD yield was higher for the latter catalyst. However, acetaldehyde and BD selectivity with respect to ethanol conversion followed opposite trends for both catalysts. These results led the authors to conclude that acetaldehyde was the BD precursor. On the other hand, both BD and ethylene selectivity were increased linearly with ethanol conversion over Ag‐exchanged aluminated sepiolite. Based on these observations, the authors concluded that Prins condensation of acetaldehyde with ethylene yielded BD (Scheme 7). The nucleophilic ethylene anchors the activated carbonyl (electrophile) site of acetaldehyde, leading to the formation of BD [29]. However, no experimental studies have confirmed the Prins condensation reaction. Moreover, omission of 3‐buten‐2‐ol introduces an uncertainty about the validity of the Prins condensation mechanism. The thermodynamic analysis indicates that 3‐buten‐2‐ol formation from acetaldehyde and ethylene condensation is highly endergonic [13]. DFT calculation further reveals that this C–C bond formation has a higher energy barrier than acetaldehyde self‐aldol condensation over MgO and ZrO_2_ catalysts, implying the latter pathway is kinetically more favorable [30, 31]. Natta and Rigamonti also overruled the Prins condensation mechanism due to the lack of improvement in BD yield when ethylene was introduced in the feed [26].

### 2.3 Mechanistic Insight by Diffuse Reflectance Infrared Fourier Transform Spectroscopy (DRIFTS) Study

The ETB mechanism has been gradually shaped by fragmented evidence accumulated over several decades, with many disagreements within the scientific community. However, modern physicochemical techniques, including in situ and DFT studies, now allow for more precise elucidation of reaction mechanisms. The absence of acetaldehyde self‐aldol condensation product, 3‐hydroxybutanal, is one such glaring issue with the Quattlebaum mechanism (Scheme 5) [32, 33, 34]. The early studies justified the absence of 3‐hydroxybutanal due to its rapid conversion to crotonaldehyde. However, subsequent investigations found that BD yield was not improved much when 3‐hydroxybutanal was cofed with ethanol [35]. Corson et al. observed that 3‐hydroxybutanal predominantly reverted to acetaldehyde rather than crotonaldehyde when an ethanol‐3‐hydroxybutanal mixture was passed over Ta/SiO_2_, contradicting the Quattlebaum mechanism [36]. However, Gao et al. identified BD, crotonaldehyde, and 3‐hydroxybutanal as key products over magnesia‐silica using mass spectrometry techniques [36]. They observed an IR band at 1717.85 cm^−1^, characteristic of C=O stretching in crotonaldehyde, which was decreased upon ethanol feeding [36]. These results suggested that crotonaldehyde reacted with ethanol to form BD. Furthermore, crotonaldehyde was in higher concentration compared to 3‐hydroxybutanal, signifying the role of 3‐hydroxybutanal as an intermediate to crotonaldehyde in the ethanol–acetaldehyde reaction [36]. Taifan et al. employed in situ temperature programmed DRIFTS of acetaldehyde using MgO–SiO_2_ (1:1) catalyst and observed an enhancement of 3‐hydroxybutanal peak at 1273 cm^−1^ while increasing temperatures above 573 K with the simultaneous decrease in vapor phase acetaldehyde peak at 1724 cm^−1^ [37]. These results confirmed the conversion of acetaldehyde to 3‐hydroxybutanal.

Müller et al. elucidated the significance of crotonaldehyde in BD formation using modulated cofeeding experiments monitored by a DRIFTS‐mass spectrometry setup over Lewis acidic Ta‐BEA catalysts [38]. The ethanol–crotonaldehyde cofeeding experiment showed a vibrational frequency of crotonaldehyde surface species at 1690 cm^–1^, likely to be involved in BD formation. These results evidenced that acetaldehyde coupling to crotonaldehyde was the likely initial step, followed by its transfer hydrogenation by ethanol to crotyl alcohol and dehydration of crotyl alcohol to BD. Their study further showed an optimal ethanol/acetaldehyde ratio of 3 to sustain a high ethoxy coverage and effectively desorb crotyl alcohol from the catalyst surface for high BD productivity. The kinetic curve over 1Ag/4ZrO_2_/SiO_2_ also showed acetaldehyde as the primary product from ethanol, with crotonaldehyde as the secondary unstable product [34, 39].

### 2.4 Molecular Mechanism

#### 2.4.1 C–C Coupling via 3‐Hydroxybutanal

**FIGURE 2:**
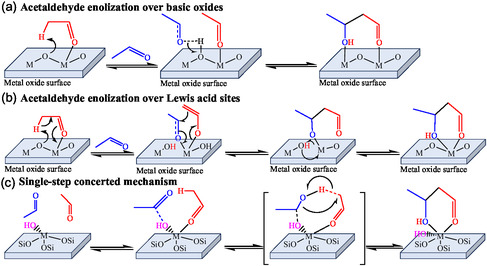
Molecular mechanism for acetaldehyde self‐aldol condensation. “M” represents the metal [13, 40, 41]. (a) Acetaldehyde enolization over basic oxides. (b) Acetaldehyde enolization over Lewis acid sites. (c) Single‐step concerted mechanism.

The current consensus is inclined toward the mechanism involving acetaldehyde self‐aldol condensation. However, the molecular mechanism is evolving based on the nature of the catalyst. The strong basic sites of alkaline‐earth metal oxides (e.g., MgO) are reported to abstract a proton from the *α*‐carbon atom of acetaldehyde to form an enolate [13]. The enolate is stabilized through electrostatic interactions with acidic sites [13]. The negatively charged *α*‐carbon atom of enolate then interacts with the *α*‐carbon of another acetaldehyde molecule to form a C–C bond. The 3‐hydroxybutanal is subsequently formed through proton back‐transfer from the catalyst (Figure 2a). On the contrary, transition metal oxides with Lewis acidity polarize the acetaldehyde carbonyl group for C–C coupling (Figure 2b) [34, 40]. However, Palangin et al. proposed an alternate mechanism based on FTIR spectroscopy and DFT calculation for BEA zeolites doped with different metals, such as Sn, Zr, and Ti [41]. They proposed a two‐step mechanism based on *α*‐proton transfer over SnBEA zeolite, facilitated by basic Si–O–Sn centers [41]. Taifan et al. also observed enolates during ethanol conversion over MgO/SiO_2_ [37]. However, DFT calculation reveals that Zr/TiBEA zeolite framework, where electronic configuration is not suitable for activation of basic sites, follows a distinct single‐step concerted mechanism (Figure 2c) [37]. This pathway involves coadsorption of two acetaldehyde molecules at the metal center and facilitates proton transfer within a collective transition state stabilized by M–OH sites, forming 3‐hydroxybutanal [37]. Although Muller et al. failed to observe enolate species using in situ IR spectroscopy, they also advocated a similar mechanism over Ta‐BEA [38]. However, their proposed mechanism differs by acetaldehyde adsorption on hydroxyl groups coordinated to metal cation or neighboring silanol groups rather than metal sites [38]. Nevertheless, these mechanisms lack evidence of surface species due to their high instability [41]. The mechanism based on DFT calculation is also limited to the specific nature of catalysts. Therefore, catalyst‐specific operando investigations, combined with advanced surface characterization, are essential to gain deeper mechanistic insights.

#### 2.4.2 Crotonaldehyde Reduction

**FIGURE 3:**
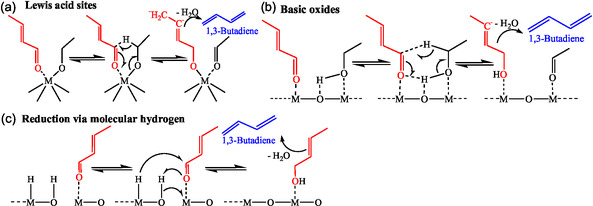
Molecular mechanism for crotonaldehyde reduction to crotyl alcohol [42, 43, 44]. (a) Lewis acid sites. (b) Basic oxides. (c) Reduction via molecular hydrogen.

Crotonaldehyde is involved in several mechanisms, including Quattlebaum, Natta, and Rigamonti. Crotonaldehyde is then converted to BD either directly or via crotyl alcohol [26]. Many researchers also recognized crotyl alcohol as an intermediate, although it was not observed in their studies, possibly due to its rapid dehydration to BD. Crotyl alcohol dehydration to BD is also thermodynamically favorable [25]. DRIFTS‐monitored temperature‐programmed surface reaction (TPSR) experiments exhibited that the disappearance of chemisorbed crotyl alcohol coincided with the detection of BD [37]. Niiyama et al. proposed that crotonaldehyde reduction ensued through an intermolecular hydrogen transfer from ethanol over acid–base pairs via the Meerwein–Ponndorf–Verley–Oppenauer (MPVO) mechanism (Figure 3) [27]. A recent study showed a drop in BD formation over Ag/ZrO_2_/SiO_2_ with increasing ethanol pressure due to competitive adsorption of ethanol on sites occupied by crotonaldehyde [45]. The reactivity pattern thus indicates that MPVO reduction likely follows a bimolecular Langmuir–Hinshelwood mechanism, requiring coadsorption of crotonaldehyde and ethanol on the same metal site (e.g., Zr) or on adjacent metal sites for nontransition metal oxides (Figure S1, Supporting Information) [42, 45]. Further validation of this mechanism was provided by deuterium labeling studies, where ethanol deuterated at the *α*‐carbon atom resulted in the formation of CD_2_‐CH=CD‐CH_2_, confirming the involvement of a six‐membered transition‐state complex following MPVO reduction [45]. Tamura et al. have conducted similar kinetics, such as an isotope effect study, to explore the crotonaldehyde hydrogenation [43]. Baba et al. recently demonstrated that crotonaldehyde reduction by ethanol did not proceed on CaO due to its inability to activate heterolytic dissociation of hydrogen [13]. They suggested an alternate mechanism, where chemisorbed hydrogen reduced crotonaldehyde to crotyl alcohol (Figure S2, Supporting Information) [13].

#### 2.4.3 Alternative Mechanism

**FIGURE 4:**
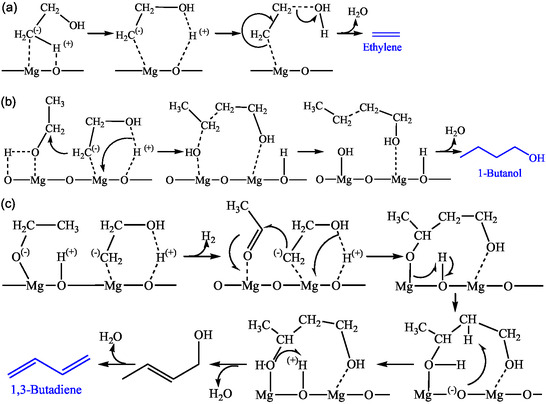
Mechanism for ethanol conversion to (a) ethylene, (b) 1‐butanol, and (c) BD.

Cavani et al. examined the involvement of 3‐hydroxybutanal in the mechanism that was popularly considered a reaction intermediate [44]. Their FTIR‐aided TPSR experiment using MgO preadsorbed with ethanol revealed that neither 3‐hydroxybutanal alone nor cofeeding with ethanol resulted in BD formation. Moreover, peaks attributed to crotyl alcohol were observed at lower temperatures than the crotonaldehyde peak [44]. These observations led them to conclude that C_4_ alcohols were kinetically preceded over their precursors. Based on in situ DRIFTS study and DFT calculation, they proposed an alternative C–C coupling mechanism via the reaction between C_2_‐oxygenated species and a surface carbanion formed by deprotonation of methyl groups of ethanol on MgO catalyst (Figure 4) [44]. This carbanion either reacts with a previously dissociated proton to form water and ethylene or attacks a neighboring ethanol or acetaldehyde molecule, generating water and either crotyl alcohol or 3‐buten‐1‐ol, both of which subsequently dehydrate to BD. This mechanism offers a unifying perspective, linking with other ethanol conversion pathways, such as the Guerbet reaction (leading to 1‐butanol) and ethylene formation [44]. However, independent studies have yet to verify this mechanism. DFT calculations found that the carbanion was unstable on MgO, favoring ethylene formation over BD [30]. Cavani et al. noted that a specific Mg/Si ratio of 15 was necessary for this pathway, suggesting that pure MgO and MgO–SiO_2_ with high magnesia content were required for this mechanism.

### 2.5 Kinetic Modeling

The rate‐determining step is another ambiguity in the overall mechanistic landscape. The rate‐determining step generally varies depending on the nature of the catalysts and reaction parameters. In the one‐step process, acetaldehyde was accumulated by reducing contact times, suggesting aldol condensation as the rate‐limiting step [27, 45]. However, crotonaldehyde formation via aldol condensation was proposed as the rate‐determining step for bifunctional metal‐promoted oxides [46]. This conclusion was based on the evidence that adding crotonaldehyde to ethanol enhanced BD formation compared to acetaldehyde addition. In contrast, recent studies using Ag/ZrO_2_/SiO_2_ reported *α*‐H abstraction from acetaldehyde in aldol condensation as the rate‐limiting step [45, 46].

The kinetic models capture the effect of key parameters on reaction rates, such as temperature, pressure, concentration, etc. The kinetic models thus play an important role in reactor design. Tretyakov et al. developed a kinetics of the one‐step process for (K_2_O)ZnO/*γ*‐Al_2_O_3_ catalyst in the presence of H_2_O_2_ initiator (Table S1, Supporting Information) [47, 48]. They observed the formation of BD, butylenes, ethylene, acetaldehyde, butanal, water, and hydrogen. Three distinctive active centers were proposed, with two being responsible for BD formation and the third being for oxygenated byproducts, such as acetaldehydes, DEE, and butanal [47]. The BD formation via acetaldehyde condensation was found to have the lowest activation energy, whereas the ethanol dehydration to ethylene and 1‐butylene dehydrogenation to BD were kinetically hindered. The study also indicated a zero‐order dependence for BD formation, competitive adsorption of acetaldehyde and butyraldehyde on the active sites, and an Eley–Rideal mechanism for ethylene dimerization [47]. However, since water adsorption was not considered in the kinetics, Tretyakov et al. model could not account for the effect of water on the performance of the one‐step catalysts [49]. Therefore, González et al. modified the power‐law kinetic model by introducing a corrective term to account for the inhibitory effect of water on the reaction rate [50]. The BD productivity was found to increase with acetaldehyde and ethanol partial pressures. On the other hand, Dussol et al. developed a kinetic model for the two‐step process using Ta_2_O_5_–SiO_2_ [51]. Multiple reaction pathways were considered in their study, including acetaldehyde aldol condensation and competing reactions for the formation of DEE, ethylene, and oxygenated byproducts (Table S2, Supporting Information). The acetaldehyde aldol condensation was found to be the rate‐limiting step, while intramolecular hydrogen transfers governed byproduct formation. Furthermore, the aldol condensation pathway exhibited the lowest activation energy [51].

Da Ros et al. performed a microkinetic analysis of the one‐step reactions over MgO–SiO_2_ catalyst based on detailed characterization of experimental fluctuations or the total variability associated with composition measurements in the reactor outlet [52]. The analysis considered the influence of temperature and catalyst properties on ethanol conversion and product selectivity. The observed results established that the variations in product selectivity were not independent of each other. Furthermore, a local microkinetic information‐contained covariance matrix indicating the experimental fluctuations was developed. Based on the change in the correlation coefficient of the covariance matrix, a change in the ethanol conversion mechanism was reported with temperature. At 723 K, the reactant–product relationship between ethanol and acetaldehyde vanishes. Simultaneously, an emergence of a reactant–product relationship between acetaldehyde and butadiene was observed above 723 K. The authors interpreted these observations as a change in the reaction kinetics. Between 573 and 673 K, aldol condensation was the rate‐determining step. However, at 723 K, ethanol dehydrogenation to acetaldehyde became kinetically relevant, influencing BD formation. At this temperature, the reactant–product relationship between ethanol and acetaldehyde weakened, while a new correlation between acetaldehyde and BD emerged [52]. These findings align with thermodynamic analysis, which suggests that higher temperatures favor acetaldehyde formation, leading to its condensation into heavier products [10]. Additionally, the correlation coefficient of hydrogen remained positive throughout the operating regime, implying that hydrogen was not involved in the crotonaldehyde reduction step as indicated by thermodynamic analyses [52].

### 2.6 Side Reactions

**SCHEME 8:**
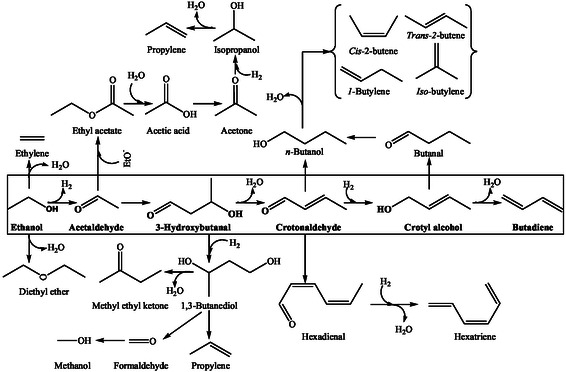
The reactions involved in the formation of byproducts and intermediates.

The main ETB reactions are accompanied by significant byproduct formation (Scheme 8) [10]. Quattlebaum et al. classified these byproducts based on their formation mechanisms: dehydration, ester‐forming disproportionation, or BD reaction cascade [24]. The dehydration produces ethylene and DEE from ethanol, while ethyl acetate is formed via acetaldehyde disproportionation (Tishchenko reaction) [20]. Acetic acid results from the hydrolysis of ethyl acetate. Other notable byproducts include *n‐*butanol via Guerbet reaction, butylenes from *n‐*butanol dehydration, propylene from acetone or ethanol‐to‐propylene pathways, and C_5_^+^ hydrocarbons by aldol condensation of crotonaldehyde [20, 34]. Methyl ethyl ketone is a byproduct formed through the rearrangement of deoxygenated 3‐hydroxybutanal or dehydration of 1,3‐BDO [53]. Butanal is another byproduct formed from crotyl alcohol. The C_6_ hydrocarbons and oxygenates are formed through condensation of C_4_ aldehydes, such as butanal and crotonaldehyde, with acetaldehyde [24].

The nature of catalysts strongly influences byproduct formation. Toussaint et al. reported the formation of ethylene, ethyl acetate, pentenes, pentadienes, acetic acid, and butanol over strongly acidic tantalum oxide on silica gel catalyst above 573 K [46]. The silica‐supported zirconium oxides, which catalyze condensation reactions, produce excessive amounts of C_6_^+^ hydrocarbons [34]. The cross‐coupling between acetaldehyde and crotonaldehyde produces hexadienal, which converts to hexatriene [24]. Besides crotonaldehyde, a host of aldehydes and ketones are also observed, originating via aldol condensation of acetaldehyde [54]. Moreover, retro‐aldol addition of 4‐hydroxybutanone leads to acetone and formaldehyde, both of which undergo aldol reactions or Meerwein–Ponndorf–Verley (MPV) reductions, forming propylene and pentadiene [24].

## 3 Thermodynamics

Thermodynamic analysis depicts the spontaneity of the reactions involved in a mechanism. This analysis also helps in identifying optimal reaction conditions to improve the equilibrium yield of the desired product. The standard enthalpy (ΔH°) and Gibbs free energy (Δ*G*°) change of the reactions involved in the mechanisms are shown in Table S3 (Supporting Information). The thermodynamic analyses showed that hydrogenation of crotonaldehyde to crotyl alcohol via MPV reaction was more favorable than using molecular hydrogen [26]. These results were validated by Bhattacharya and Sanyal [55]. The apparent activation energy of BD formation from the ethanol–crotonaldehyde mixture was found to be 15.3 kcal · mol^−1^, which was lower in comparison to that for BD formation from pure ethanol and crotonaldehyde–hydrogen mixture [55]. Moreover, theoretical and experimental studies established that crotonaldehyde formation by acetaldehyde self‐aldol condensation was the rate‐limiting step, contrary to the widely assumed crotonaldehyde reduction to crotyl alcohol at that time [55]. Though self‐aldol condensation of acetaldehyde is widely accepted C–C coupling reaction, however, our thermodynamic analysis exhibited a positive Δ*G*° at 298 K, and it was increased with rising temperatures, implying its nonspontaneity [25]. These results suggest that the feasibility of aldol‐condensation hinges on the rate of subsequent 3‐hydroxybutanal‐consuming reactions. Our analysis further demonstrates that acetaldehyde C–C coupling to crotonaldehyde has a somewhat positive Δ*G*° value in the entire temperature range. Notably, all proposed reactions are spontaneous in the Prins condensation mechanism [25]. However, this mechanism lacks experimental evidence.

**FIGURE 5:**
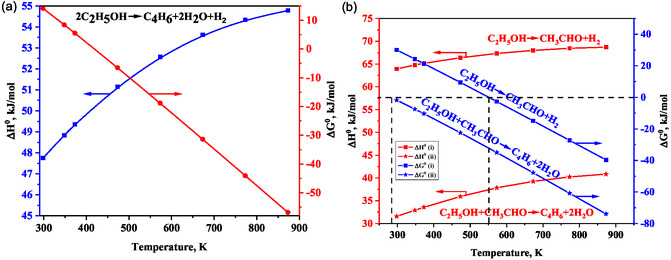
Δ*G*° and Δ*H*° of overall (a) one‐step and (b) two‐step ETB reactions.

Bhattacharyya and Ganguly reported the endothermic nature of the overall one‐step reaction, with a substantial increase in the equilibrium constant at elevated temperatures [56]. Our thermodynamic analysis showed a positive Δ*G*° for this reaction below 417 K, and it became spontaneous above 417 K [25]. Angelici et al. also confirmed the spontaneity of the overall one‐step reaction above 423 K [20]. Bhattacharyya and Avasthi further reported that the overall reactions in the two‐step process were feasible between 298 and 723 K [57]. The first step of the two‐step process is spontaneous above 550 K, while the second step is spontaneous in the entire temperature range above 298 K (Figure 5) [25]. Besides, the Δ*G*° value was more negative for the second step of the two‐step process than the overall one‐step reaction.

Makshina et al. recently calculated equilibrium product distribution in the temperature range of 298–803 K based on the Toussaint–Kagan mechanism using Aspen Plus [10]. Their investigation highlighted the thermodynamic bias toward secondary reactions, with lower overall BD selectivity than the experimental results [12]. Our thermodynamic analysis also showed the low equilibrium BD selectivity for both one‐step and two‐step processes, which was enhanced at low pressure and high temperatures [25]. However, our results showed that the equilibrium BD selectivity was relatively higher in the two‐step process than in the one‐step counterpart. The highest equilibrium BD selectivity was observed at 1:1 ethanol/acetaldehyde mole ratio [25]. Liu et al. calculated the equilibrium product composition across the temperature range of 473–1273 K, considering numerous potential intermediates and byproducts [58]. They observed the minimal formation of acetaldehyde at lower temperatures (<573 K), with methane, CO, and CO_2_ being more favorable [58]. Similarly, Garbarino et al. and Banu et al. also observed a low yield of acetaldehyde at 373–773 K due to its thermodynamic limitations, favoring its decomposition to methane, CO, and other reactions, such as those producing acetone and propylene [59, 60].

## 4 Trends in Catalytic Systems

The catalyst compositions developed during the late 19th century were undisclosed despite successful BD production from ethanol. Thus, extensive research efforts have been dedicated to developing catalysts for the ETB reaction. Since ethanol dehydrogenation to acetaldehyde was well‐established at the time, much of the early research was focused on developing catalysts to convert an ethanol–acetaldehyde mixture into BD. The reaction was known to follow a complex pathway, comprising dehydration, dehydrogenation/hydrogenation, and C–C coupling. Therefore, an ideal ETB catalyst must be multifunctional with an optimal combination of different catalytic sites needed for individual reactions, such as acidic, basic, redox, metal, etc. The most commonly employed catalysts were mixed oxides with redox properties, such as MgO–SiO_2_ [61, 62, 63], ZrO_2_–SiO_2_ [64, 65, 66], and their derivatives obtained through modification by metals, such as Ag [67, 68], Cu [69, 70], Ta [69], and Au [71]. Metal modification promotes ethanol dehydrogenation and C–C coupling reactions. Further studies were focused on modifications using metal oxides, such as ZrO_2_ [58, 72], ZnO [72, 73, 74, 75], In_2_O_3_ [76], and Ga_2_O_3_ [77]. This section provides a comprehensive overview of the trends in catalytic systems for both one‐step and two‐step processes. The catalyst deactivation is also reviewed to obtain tangible insights into challenges toward commercialization.

### 4.1 Magnesium‐Based Catalysts for One‐Step ETB Process

Since the one‐step process was invented first, numerous catalysts with acidic, basic, or their combination have been studied extensively.

#### 4.1.1 MgO–SiO_2_ Catalysts

The earliest work toward the one‐step process was carried out by Sergey Lebedev in 1903, with considerable amounts of BD produced over metal–oxide catalysts, comprising both dehydration and dehydrogenation activity [20]. Since the Lebedev catalyst was guarded by patents, several studies emerged around the same time, such as by Natta and Rigamonti [26] and Corson et al. [78]. They compared the BD yields from their test runs with historical data of Lebedev and revealed that the catalyst was comprised of mixed oxides, mostly silicon and magnesium oxides, with small amounts of other oxides as promoters [20]. It was hypothesized that magnesium oxide, being basic, initiated ethanol dehydrogenation and aldol‐condensation [70, 79]. On the other hand, silica generates weakly acidic hydrous magnesium silicates containing Mg−O−Si bond, which promotes dehydration activity. The magnesia‐silica catalysts have thus been studied extensively by various research groups ever since. They are an effective choice for the one‐step process and the basis for developing industrial catalysts.

In one of the earliest studies, Kvisle et al. reported the performance of silica‐supported MgO catalysts prepared by the coprecipitation method (Table 1) [85]. The catalysts revealed good performance at a relatively low temperature of 623 K, with steady‐state ethanol conversion of almost 55%, with 30% BD and 55% ethylene selectivity [85]. The ethanol conversion and yield of BD and ethylene were increased by rising temperatures and reducing ethanol feed rates [85]. However, acetaldehyde became dominant at elevated temperatures and space velocity. Further studies revealed that the composition of MgO–SiO_2_ strongly influenced BD selectivity. Multiple studies reported a volcanic‐type trend of BD yield with MgO content [79, 80]. The observed trend was associated with the interplay between SiO_2_ and MgO phases [80]. The MgO increases both basicity and acidity via Mg–O–Si bonds. On the other hand, silica improves the surface area, with better dispersion of the active sites [80]. However, increasing silica content beyond an optimum negatively impacts the surface area and pore volume due to the partial coverage of the external MgO surface by SiO_2_ particles, resulting in loss of catalytic activity [80]. Shylesh et al. observed the maximum BD yield at around 1:1 MgO/SiO_2_ molar ratio (or a weight ratio close to 0.67) [71]. Chung et al. observed a similar volcano‐like correlation of MgO content with catalyst performance [86]. Ohnishi et al. further verified the 1:1 MgO/SiO_2_ mole ratio as the optimum for best catalytic performance [81]. However, Niiyama et al. reported a maximum BD selectivity at 85 mol% MgO content [27]. Further increase in MgO content had detrimental effects on BD selectivity due to greater concentration of metallic sites and enhanced basicity, leading to activation of undesirable side reactions [27]. In contrast, Makshina et al. reported minimal variation in ethanol conversion and BD selectivity across different Mg dispersions on silica [82]. However, a pronounced decline in BD selectivity, from 30% to 50% to approximately 20%, occurred when tetraethyl orthosilicate was employed as the silica precursor [82]. The reduction in BD selectivity was attributed to the formation of a less defective, weakly acidic SiO_2_ structure, which promoted ethanol dehydration to ethylene at the expense of the acid–base‐mediated cascade reactions essential for BD formation [82]. These results suggest that the acidic component, namely the SiO_2_ phase, plays a crucial role in BD selectivity. Nevertheless, further studies are required to substantiate this claim.

**TABLE 1:** Catalytic performance of MgO–SiO_2_ catalysts in the one‐step conversion of ethanol to 1,3‐butadiene.

SN	Catalysts	**WHSV** (h^−1^)	T (K)	P (bar)	Gas (ml/min)		X (%)	Selectivity (%)			References
					H_2_	N_2_		BD	E	Ac	
1	0.83 Mg–Si	8^a^	623	1	18	18	53	30	53	10	[80]
		16^a^					32	28	49	16	
		32^a^					22	25	51	15	
	0.63 Mg–Si	8					62	24	65	7	
2	10 wt% MgO–SiO_2_	1.1	573	1	0	20^b^	10	<5	80	<15	[72]
	30 wt% MgO–SiO_2_						~10	~5	75	15	
	50 wt% MgO–SiO_2_						~5	~15	~60	~30	
	80 wt% MgO–SiO_2_						<5	>15	~45	~45	
3	0.2 mol% Mg–Si	0.6	673	1	0	30	~35	~20	~15	<5	[81]
	0.5 mol% Mg–Si						~65	~65	~28	~5	
	1.0 mol% Mg–Si						~20	<2	~10	~10	
4	1:1 Mg–Si	—	623	1	—	—	50	84	—	—	[82]
	3:1 Mg–Si		653				54	62	—	—	
5	50 mol% MgO	—	653	0.1	—	—	—	~0.1^c^	~1.8^c^	—	[27]
	85 mol% MgO						—	>2^c^	~1^c^	—	
	100 mol% MgO						—	~0.2^c^	<0.1^c^	—	
6	MgO–SiO_2_–a	1.5 (vol%) in Ar	623	1	0	20^d^	—	~35	—	—	[83]
	MgO–SiO_2_–b						—	~30	—	—	
	MgO–SiO_2_–c						—	~20	—	—	
	MgO–SiO_2_–d						—	>45	—	—	
	MgO–SiO_2_–e						—	~40^e^	—	—	
7	MgO–SiO_2_ f	—	723	1	0	50	~68	~62	~20	~8	[84]
	MgO–SiO_2_ g						~45	~42	~38	~9	
	MgO–SiO_2_ h						~38	~28	~48	~18	
8	MgO–SiO_2_ i	3.0 g_ethanol_ g_cat_^−1^ h^−1^	673	1	15 (v/v)% Et/He		50	~60	~15^j^	<5	[62]
	MgO–SiO_2_ k						50	<5	>90^j^	<5	

X, Conversion; Et, Ethanol; E, Ethylene; DEE, Diethyl ether; Ac, Acetaldehyde; Ct, Crotonaldehyde; MgOSiO_2_–a, Prepared by mechanical mixing of calcined Mg and Si oxides; MgO–SiO_2_–b, Mechanical mixing of MgOH and silica; MgO–SiO_2_–c, Prepared by wet‐kneading method MgOH and silica (TEOS precursor); MgO–SiO_2_–d, Prepared by excess solvent impregnation, and MgO–SiO_2_–e, Prepared by wet‐kneading MgO and silica in ethanol solvent.

a Feed rate in μL h^−1^.

b He carrier gas.

c Unit is 10^−5^ mol m^−2^ h^−1^.

d Argon carrier gas.

e Mg/Si ratio = 2.

f Flower‐like.

g Nanodisk.

h Nanosheets.

i Internally hydrolyzed sample containing high specific surface area MgO. Selectivity: C_4_ hydrocarbons: ~20%, Others: <2%.

j DEE + E.

k Internally hydrolyzed sample containing low specific surface area MgO. Selectivity: C_4_ hydrocarbons: <2%, Others: <1%.

The catalytic performance also depends on the structural and textural properties of MgO–SiO_2_. Li et al. investigated the influence of MgO precursor morphologies (flower‐like, nanodisk, and nanosheet) on BD selectivity [83]. They demonstrated that the flower‐like MgO precursor achieved the highest BD yield, with minimum formation of ethylene [83]. The catalyst characterization revealed that the unique structure of the MgO precursor enhanced the Lewis basicity at Mg–O–Si interfacial sites due to the stronger interactions between MgO and SiO_2_ species compared to other morphologies [83]. The flower‐like structure also exhibited superior textural properties, such as wider pores and higher pore volume, facilitating easier reactant/product diffusion and accessibility of active sites [83]. The catalyst preparation method also plays a decisive role in catalytic performance. One of the earlier studies demonstrated that wet‐kneaded MgO–SiO_2_ outperformed the catalysts prepared by mechanically mixed counterpart due to the homogeneous dispersion of active components during the wet‐kneading [85]. The synergetic interactions between the active sites are thus significant in enhancing the catalytic performance [85]. These observations align well with Natta and Rigamonti's work on the kneading liquid [26]. The catalysts prepared in water exhibited enhanced catalytic activity compared to those kneaded in ethanol, owing to improved dissolution of SiO_2_ and MgO, leading to balanced acid–base site distribution [26].

A recent study suggested that high surface area MgO–SiO_2_ exhibited superior ethanol coupling and dehydrogenation activity, likely due to a higher concentration of low‐coordinated O^2−^ anions (under‐coordinated, electron‐rich oxygen species at edges or defects that enhanced Lewis basicity) [61]. These studies thus underscore the importance of structural, textural, and compositional optimization in designing efficient MgO–SiO_2_ catalysts. However, MgO–SiO_2_ catalysts are facing several challenges, including insufficient catalytic efficacy and stability [77]. Ochoa et al. reported significant byproduct formation (ethylene and heavier hydrocarbons like aromatics) at elevated temperatures, leading to coke deposition and rapid deactivation [77]. Moreover, structural degradation via phase transformation (inactive magnesium silicate formation) and MgO sintering were reported at elevated temperatures, diminishing active sites [77]. Metal modification of MgO/SiO_2_ has thus emerged as a promising strategy to moderate acidity, prevent phase segregation, and maintain active site accessibility [48].

#### 4.1.2 Metal‐Modified MgO–SiO_2_

The modification of MgO–SiO_2_ with metal promoters, such as Ag [67], Au [71], Cu [67], Ga [77], etc., is a promising strategy to enhance dehydrogenation activity (Table 2). Makshina et al. demonstrated that metal‐doping via impregnation increased ethanol conversion and BD yield [82]. Among the tested dopants, Cu, Zn, and Ag exhibited the highest catalytic activity, with enhanced ethanol conversion and increased BD productivity [82]. However, Zn‐doping promoted reforming reactions, forming methane, CO_*x*_, and C_4_ oxygenates, with a slightly reduced BD selectivity compared to Cu and Ag dopants [82]. In contrast, Mn‐doping showed the lowest catalytic activity [82].

**TABLE 2:** Catalytic efficacy of metal‐modified MgO–SiO_2_ catalysts in the one‐step conversion of ethanol to 1,3‐butadiene at 1 bar.

SN	Catalyst	Feed	T (K)	N_2_ (ml/min)	X (%)	Selectivity (%)						References
						BD	E	Ac	DEE	B	Others	
1^a^	MgO–SiO_2_	Et^b^	623	20^d^	37	39	32	—	—	—	—	[83]
	Ag/MgO–SiO_2_				97.1	58	6	—	—	—	—	
	Cu/MgO–SiO_2_				97.5	60	6	—	—	—	—	
	Zn/MgO–SiO_2_				99.1	53	6	—	—	—	—	
	Mn/MgO–SiO_2_				41.1	53^e^	14	—	—	—	—	
	Ag/MgO–SiO_2_	Et^c^			61.1	55	5	—	—	—	—	
	Cu/MgO–SiO_2_				47.6	56	6	—	—	—	—	
	Zn/MgO–SiO_2_				65.2	54	6	—	—	—	—	
	Ag/MgO–SiO_2_		673		91.8	54	12	—	—	—	—	
	Zn/MgO–SiO_2_				98.7	46	8	—	—	—	—	
2	MgO–SiO_2_	Et^f^	698	420^d^	30	9.2	14.7	1	3.6	0.3	0.6	[68]
	1% Cu/MgO–SiO_2_				57.5	28.2	20.1	5.6	0.9	4.3	0.6	
	1% Ag/MgO–SiO_2_				57	30.2	17.1	4	0.9	3.7	1.1	
3	MgO–SiO_2_	Et	673	7	65.9	49.3	26.7	—	—	—	—	[78]
	1% Ga/MgO–SiO_2_				99.9	34.6	28.8	—	—	—	—	
	5% Ga/MgO–SiO_2_				98.8	53.1	15.7	—	—	—	—	
	7% Ga/MgO–SiO_2_				99.7	42.2	13.3	—	—	—	—	
4	3% Au/MgO–SiO_2_	Et	480	30^g^	~10	~55	<1	~38	—	~5	—	[72]
			540		~35	~60	<5	~30	—	>5	—	
			573		~45	>60	~5	~25	—	~5	—	
4	2.5%Cu–2.5%Ag/MgO–SiO_2_ h	Et	598	20	~60	~35	—	~10	—	—	—	[69]
	2.5%Cu–2.5%Ag/MgO–SiO_2_ i				>60	~40	—	~10	—	—	—	
	2.5%Cu–2.5%Ag/MgO–SiO_2_ j				~50	~30	—	~15	—	—	—	
5	ZrZn/MgO–SiO_2_	Et	648	—	40	35.9	32.2	8.3	9.8	9.2	—	[73]
	2% Na/ZrZn/MgO–SiO_2_				24	48.5	15.5	17.3	3.6	9.8	—	
	1.2% K/ZrZn/MgO–SiO_2_				22	51.6	17.4	13.7	5.2	7.5	—	

X, Conversion; Et, Ethanol; E, Ethylene; DEE, Diethyl ether; Ac, Acetaldehyde, and B, Butylene. All metal loadings are in wt%.

a M/Si = 0.05 and Mg/Si = 2.

b Et concentration: 1.5 × 10^4^ ppm.

c Et concentration: 5.7 × 10^4^ ppm.

d Ar carrier gas.

e Low BD productivity.

f 80 vol% aqueous ethanol.

g He carrier gas.

h Conventional,.

i Microwave‐assisted.

j PVA/NaBH_4_‐assisted catalyst preparation.

Bimetallic catalysts offer enhanced catalytic activity and stability compared to monometallic counterparts owing to their synergistic interaction [68]. The Ag/Cu‐doped MgO–SiO_2_ significantly enhanced BD yield and byproducts, such as acetaldehyde, ethylene, etc [69]. It could be attributed to the evolution of new active sites for ethanol dehydrogenation and [Mg–O–Cu(Ag)] acid–base. These new sites enhanced aldol condensation, forming BD, butylenes, and ethanol dehydration products. However, Cu‐doping favored ethylene formation compared to Ag [67]. The experimental results indicated that Ag modification was more beneficial for a higher BD yield [67]. However, Ag/MgO–SiO_2_ suffered from rapid deactivation due to carbon deposition [68]. Alkali modification (Li, Na, and K) was thus investigated to improve BD selectivity by suppressing the formation of ethylene and DEE [72]. However, ethanol conversion decreased further due to neutralization of acidic sites [72]. Similarly, other bimetallic catalysts, such as Zr–Zn/MgO–SiO_2_, were explored in recent times, where homogeneous dispersion of Zr^4+^/Zn^2+^ sites ensured balanced acid–base functionality, achieving 65% BD selectivity [72]. Ethanol conversion was, however, low due to the lack of strong acid sites (Table 2) [72].

Recent studies highlighted the role of gallium (Ga) as a promoter [77]. Ga doping introduces Ga–O(H)–Si sites with moderate Lewis acidity, which enhances ethanol dehydrogenation and acetaldehyde conversion to BD via crotyl alcohol. The Ga loading also affected the acidity, and the Ga loading above an optimal level enhanced acidity, leading to increased ethylene formation [77]. The Au/MgO–SiO_2_, another promising catalyst, achieved superior BD yield (>60% at 573 K) with minimal byproducts (~5% ethylene) [71]. Their study further demonstrated the lower ethanol conversion and BD selectivity over Au‐doped magnesia‐silica containing the bulk MgO phase, indicating the significance of proper metal dispersion for enhanced catalytic activity [71].

#### 4.1.3 Metal‐Oxide Modified MgO–SiO_2_

Metal oxides, such as ZnO [84], Cr_2_O_3_ [78], In_2_O_3_ [76], etc., are doped on MgO–SiO_2_ to improve catalytic performance (Table 3). The ZnO was reported to passivate strong Lewis acid sites and weaken Brønsted acid sites while simultaneously introducing basicity [87]. It also exhibited excellent performance in ethanol dehydrogenation [90]. Consequently, ZnO‐modified MgO–SiO_2_ has been widely investigated in recent years. Sekiguchi et al. reported enhanced BD production rates over Zn‐containing talc catalysts [84]. Wang et al. demonstrated that ZnO doping led to the formation of highly dispersed ZnO species, thereby enhancing ethanol dehydrogenation activity and BD yield due to the stronger surface basicity in the Mg–O–Si interfacial structures [74]. However, the product distribution depends on the Zn‐loading [74]. The low ZnO doping exhibited improved efficacy for C–C coupling with exceptional BD selectivity [74]. However, the higher ZnO loadings disrupted the Mg–O–Si interfacial structures, suppressing C–C coupling and shifting the reaction toward ethanol dehydrogenation.

**TABLE 3:** Catalytic performance of metal oxide‐modified MgO–SiO_2_ catalysts for the conversion of ethanol to 1,3‐butadiene at 1 bar.

SN	Catalyst	**WHSV** (h^−1^)	**T** (K)	N_2_ (ml/min)	**X** (%)	Selectivity (%)						References
						BD	Ac	E	DEE	Bu	Others	
1	MgO–SiO_2_ a	8.4	673	500	11.8	25.8	—			—	—	[87]
	1.4% ZnO–MgO–SiO_2_ b				43.2	49.4	23.7	8.3	7.3	1.1	10.2^c^	
2^d^	MgO–SiO_2_	1.63	723	20	45.6	46.2	20.9	20.7	0.3	—	11.9	[75]
	0.1% ZnO/MgO–SiO_2_				62.3	47.7	23.5	15.8	1.1		11.9	
	0.4% ZnO/MgO–SiO_2_				81.6	43.7	35.1	6.8	1.5	—	12.9	
	0.8% ZnO/MgO–SiO_2_				82.4	37.3	45.6	4.3	1.0	—	11.8	
	5% ZnO/MgO–SiO_2_				90.7	10.7	72.5	3.8	1.5	—	11.5	
	0.2% ZnO/MgO–SiO_2_		573		2.2	5.6	74.6	5.6	3.6	—	10.6	
	0.2% ZnO/MgO–SiO_2_		673		27.2	42.7	36.8	8.7	1.7	—	10.1	
	0.2% ZnO/MgO–SiO_2_		723		80.5	57.7	20.4	8.5	0.8	—	12.6	
	0.2% ZnO/MgO–SiO_2_		773		99.1	51.0	15.4	15.9	0.3	—	17.4	
3^e^	ZnO/MgO	1	648	‐f	10	~10	~68	~20		—	—	[88]
	ZnO/MgO–SiO_2_ (3:1)				~34	~64	24	~10		—	—	
	ZnO/MgO–SiO_2_ (1:1)				~56	~62	~22	~10		—	—	
	ZnO/MgO–SiO_2_ (1:3)				~50	~52	~26	~18		—	—	
	ZnO/SiO_2_				~48	~16	~54	~30		—	—	
4	Al–MgO–SiO_2_ (1:1:1)	0.25^g^	523	25^f^	<5	–	~70	~5	~25	—	—	[89]
			623		~7	<5	~65	~10	~15	<5	<5	
			723		>38	~10	~40	~25	~10	<5	<5	
5^h^	ZnO/MgO–SiO_2_ (3:1)	1^i^	698	7.5^f^	38.6	61.7	15.7	12.5		—	—	[76]
	ZnO/MgO–SiO_2_ (1:1)				44.8	56.2	14.8	20.8		—	—	
	ZnO/MgO–SiO_2_ (1:3)				38.3	37.2	21.0	38.8		—	—	
6^j^	MgO–SiO_2_	0.5^i^	698	20^k^	>95	~15	<2	~65	<2	<2	—	[77]
	2% In_2_O_3_/MgO–SiO_2_				>95	~45	~5	~40	~10	<5	—	
	5% In_2_O_3_/MgO–SiO_2_				~95	~45	~13	~20	~5	~12	—	
	10% In_2_O_3_/MgO–SiO_2_				~90	~30	~35	~10	<5	~10	—	

X, Conversion; Et, Ethanol; E, Ethylene; DEE, Diethyl ether; Ac, Acetaldehyde; Bu, Butanol, and B, Butylene. All metal loadings are in wt% basis.

a MgO‐SiO_2_ prepared by kneading magnesium hydroxide and colloidal silica.

b 34.1 wt% MgO and 63.6 wt% SiO_2_.

c Others include butylenes, butyraldehyde, crotonaldehyde, CO, CO_2._.

d Others include propylene, acetone and butylenes.

e 4 wt% ZnO loading.

f Ar carrier gas.

g GHSV in cm^3^ mg^−1^ min ^−1^.

h Ethanol–water feed.

i WHSV in g_ethnaol_ g_cat_^−1^h^−1^.

j Catalysts are prepared by one‐pot synthesis method.

k He carrier gas.

The MgO/SiO_2_ ratio also plays a significant role. The ethanol conversion was reduced at high MgO content with little variation in product selectivity [91]. It may be attributed to the reduced contact surface between MgO and silica, poor Zn dispersion due to decreased silica gel content and lower surface area [91]. Conversely, increasing SiO_2_ content enhanced dehydration products, attributed to the enrichment of Lewis acidity comprising Zn and Si species [91]. These findings indicate that balancing acidic and basic sites is essential for efficient ethanol conversion to BD. The influence of acid–base properties was further verified using Mg–Al double oxides coprecipitated with SiO_2_ [88]. The catalyst with the highest medium‐density basic sites demonstrated maximum BD selectivity [88]. n‐Butanol, ethylene, and DEE were other significant products [88]. Kyriienko et al. investigated the effect of water in ethanol feed over ZnO/MgO–SiO_2_ [75]. The maximum BD selectivity from ethanol–water was observed over ZnO/MgO–SiO_2_ with the highest MgO content [75]. Chemisorption of water occurred preferentially on Mg‐containing sites, resulting in correlated changes in the acidity, notably the conversion of Lewis acid sites to Brønsted acid sites [75]. The C–C coupling products were reduced in the presence of water, likely due to the competitive adsorption of water on active sites responsible for the aldol condensation of acetaldehyde [75]. However, hydrated sites formed through the interaction with Mg and Si oxides remained catalytically active for C–C coupling even in the presence of water [75]. These findings underscore the importance of hydrophilic properties and acid–base balance to optimize BD selectivity from aqueous ethanol feed.

The structure and morphology of the catalyst also play a crucial role in the nature and density of active sites, coke resistance, and prolonged stability, complementing the compositional advantages of metal oxide modification. Szabó et al. studied the reaction using In_2_O_3_‐promoted MgO–SiO_2_ [76]. The unmodified MgO–SiO_2_ showed DEE and ethylene as the major products at lower and higher reaction temperatures, respectively, due to the inherent acidity arising from Mg–O–Si linkages [76]. The incorporation of In_2_O_3_, however, significantly enhanced dehydrogenation activity with increased formation of acetaldehyde, suppressing the formation of dehydration products: ethylene and DEE [76]. The SBA‐15 as a SiO_2_ source introduced a hexagonally ordered mesoporous structure with a high concentration of silanol groups [76]. It facilitated the high dispersion and stabilization of the catalytically active MgO and In_2_O_3_ species within the pores, providing beneficial structures for enhanced BD formation [76]. Moreover, ethylene and DEE were suppressed by increasing In_2_O_3_ loading with a corresponding decrease in BD selectivity, possibly due to the increased basicity, limiting the acetaldehyde coupling [76]. Corson et al. evaluated a range of supported mixed‐oxide catalysts, which were widely investigated during the early stages of ETB catalyst development [78].

#### 4.1.4 Ordered Catalysts

The textural properties of catalysts also play a crucial role in the ETB reaction. Well‐defined porous structures with wide pores facilitate access of reactants, intermediates, and products to and from the active sites. The synergy and balance among different catalytic sites are the key factors in the complex reaction network. These parameters were adjusted by the catalyst morphology and active site composition [83]. Therefore, Zn‐Y [89], H‐*β* [92], and metal‐modified mesoporous silica [93] have been investigated (Table 4). Dai et al. investigated the confinement effect of zeolite pore structures [89]. *β*‐Zeolite confined bicomponent Zn‐Y clusters demonstrated significant BD selectivity (~63%) under relatively mild reaction conditions [89]. The experimental results further suggested that the proximity of functional sites within zeolite channels enabled efficient transfer of acetaldehyde between active sites. This proximity facilitated the conversion of acetaldehyde into 3‐hydroxybutanal, minimizing unwanted byproduct formation [89]. However, the confinement effects of BEA zeolites showed reduced ethanol conversion despite comparable BD selectivity with ZrO_2_–SiO_2_ [95]. These findings suggest that research should focus on zeolite structures with different morphologies that can provide sufficient acidity to achieve high ethanol conversion. Klein et al. examined the influence of structural parameters in H‐*β* zeolites during two‐step ETB reactions [92]. They introduced mesoporosity to enhance the inherently low activity of unmodified H‐*β* catalysts via desilication [92]. However, the desilication led to pore collapse, resulting in diminished catalyst activity and a notable drop in BD selectivity [92]. These observations aligned with the findings of Chae et al., who studied a two‐step ETB conversion over Ta‐doped ordered mesoporous silica (OMS) [93]. Their findings indicated that increasing mesopore size led to improved ethanol conversion and BD selectivity, although excessively large mesopores caused a decline in selectivity due to reduced surface area [93]. Interestingly, comparable activity was observed between large‐pore Ta/SBA‐15 and smaller‐pore Ta/MMS, attributed to the latter's higher external surface area. The authors concluded from their results that crystal size, rather than pore size, might be a more critical parameter in optimizing the catalytic performance of OMS [93]. Ternary Ag/ZrO_2_/SBA‐16 also showed promising performance under relatively mild reaction conditions [94]. Enhanced SiO_2_ surface area facilitated better Ag dispersion, which improved ethanol dehydrogenation activity, while the Lewis acid site was proliferated due to ZrO_2_ incorporation [93]. These factors were responsible for enhanced ethanol conversion and overall catalytic performance.

**TABLE 4:** Catalytic performance of ordered catalysts for the one‐step transformation of ethanol to 1,3‐butadiene at 1 bar.

SN	Catalyst	**WHSV** (h^−1^)	**T** (K)	**N**_**2**_ (ml/min)	**X** **(%)**	Selectivity (%)								References
						BD	Ac	E	DEE	Ct	B	Bu	Others	
1	5% Zn/*β*	1.3	623	20	81	33	33	14	4	5	—	—	—	[92]
	5% Zn‐5% Y/*β*				89	69	4	8	2	1	—	—	—	
	2% Zn‐8% Y/*β*				92	74	1	9	3	1	—	—	—	
2	Ag‐1.3% ZrO_2_/BEA (19)	1.2	593	—	14.3	62.2	—	—	—	8.1	10.2	5	14.5^1^	[94]
3	Ta/SBA‐15‐60	1	523	5	31.4	72.8	—	8.7	—	0.6	1.5	—	16.5^a^	[95]
	Ta/SBA‐15‐100				46.9	79.8	—	4.5	—	1.0	2.0	—	12.7^b^	
	Ta/SBA‐15‐130				47.1	79	—	4.5	—	1.1	1.7	—	13.6^c^	
	Ta/MMS				40.7	77.9	—	4.3	—	0.8	1.8	—	15.2^d^	
4^e^	SiO_2_‐646	0.23	598	‐f	7.0	0	69.6	26.4	2.2	—	0	—	1.8	[96]
	4% ZrO_2_/SiO_2_				14.8	0	2.6	47.9	46	—	0.4	—	1.8	
	4% Ag/SiO_2_				88.3	0.9	61.4	1.5	0.1	—	5.9	—	17.3	
	4% Ag‐4% ZrO_2_/SiO_2_				89.2	73.6	6.7	5.5	3	—	5.2	—	6	
5	Al–C	4.73	648	‐g	70.1	6.2	64.6	23.3	5	—	—	0.5	4.6	[97]
	1% Zr/Al–C				78.6	30.1	3.5	12.3	5.0	—	—	0.5	8.6	
	10% Zr/Al–C				90.3	58	9.8	15.4	3.3	—	—	2.4	11.1	
	30% Zr/Al–C				44.6	15.3	20	32	11.2	—	—	0	21.5	
6	YPO_4_	2	623	28	~50	~5	—	~78	~20	—	—	—	—	[98]
	1% Co‐YPO_4_				~60	~60	<5	~15	<5	—	—	—	<5	
7^f^	Ag/SiO_2_	Et	573	‐g	26	—	97	3		—	—	—	—	[99]
	2% Ta/SiO_2_				15	12	75	8		—	—	—	5	
	4% Ta/SiO_2_				20	19	65	14		—	—	—	2	
	10% Ta/SiO_2_				21	24	50	24		—	—	—	2	
	2% Y/SiO_2_				21	38	37	6		—	—	11	8	
	4% Y/SiO_2_				21	39	35	8		—	—	11	7	
	10% Y/SiO_2_				22	39	20	13		—	—	17	11	
	2% Ta/H‐*β*				30	9	61	28		—	—	0	3	
	2% Y/H‐*β*				27	44	33	9		—	—	4	10	
	2% Ta/SiO_2_				70	70	~18	~5		—	—	—	~10	
	2% Y/SiO_2_				~60	~50	~20	~10		—	—	<5	~20	
8	1% Cu‐2% Zn‐5% Y/SiBEA	1	623	20	~60	~62	~5	~5	~10	—	—	—	~18^h^	[100]
					~90	74	<5	~8	~5	—	—	—	~20^h^	
					>95	~65	<5	~10	<2	—	—	—	~18^h^	
9	0.5% Na‐4% Ag‐4% ZrO_2_/SBA‐16	11^i^	—	598	—	89.8	75.1	2.1	—	3.3	1.8	10.2	6.9^2^	[101]
	0.5% K‐4% Ag‐4% ZrO_2_/SBA‐16					86.6	61.7	8.1	—	4.5	3.9	8.2	13.3^3^	
10	MgO–SiO_2_	0.4 (3:1)^j^	623	40	27.1	26.2	—	30.9	2.8	—	—	3.8	39.2^k^	[71]
	1.0% Ta/MgO–SiO_2_				62.6	79.6	—	5.9	1.0	—	—	1.0	12^k^	
	1.5% Ta/MgO–SiO_2_				77.5	81.9	—	6.2	1.0	—	—	0.6	9.4^k^	
	2.5% Ta/MgO–SiO_2_				82.1	74.6	—	10.3	1.2	—	—	0.7	11.6^k^	
	3.0% Ta/MgO–SiO_2_				72.7	77.6	—	7.5	1.8	—	—	1.9	10.5^k^	
11	1% Ac‐2% Ta‐3% Cu/SiO_2_	1.1	598	20	57.2	52.1	42.8	<1	—	—	—	—	<1	[102]
	1% Ac‐3% Ta‐4% Cu/SiO_2_				73.4	49.7	43.1	<1	—	—	—	—	<1	
	1% Ac‐3% Ta‐4% Cu/SiO_2_				54.9	70.7	20	3.9	—	—	—	—	5.5	
	1% Ac‐4% Ta‐4% Cu/SiO_2_				60.1	73.5	16.4	1.3	—	—	—	—	8.8	

X, Conversion; Et, Ethanol; E, Ethylene; DEE, Diethyl ether; Ac, Acetaldehyde; Ct, Crotonaldehyde; B, Butylene, Bu, Butanol, and OMS, Ordered mesoporous silica. All metal loadings are in wt%.

^f^24.3% ethanol in N_2_. ^g^~6 vol% ethanol/N_2_.

a Propylene: 1.9, Ethoxy ethane: 6.4, Ethyl acetate: 1.8, Acetic acid: 0.4, Others: 6.

b Propylene: 1.8, Ethoxy ethane: 4.0, Ethyl acetate: 1.1, Acetic acid: 0.2, Others: 5.8.

c Propylene: 1.7, Ethoxy ethane: 3.6, Ethyl acetate: 1.1, Acetic acid: 0.2, Others: 7.

d Propylene: 1.8, Ethoxy ethane: 3.5, Ethyl acetate: 1.3, Acetic acid: 0.2, Others: 8.4.

e Davisil 646 silica gel as SiO_2_ source.

f Catalysts are physically mixed with Ag‐SiO_2_.

g 6 mol% ethanol/N_2_.

h Propylene: <5, Butylene: ~5, Others: ~10.

i Unit is g_ethanol_ g_cat_^−1^ h^−1^.

j Value in () represent ethanol to acetaldehyde ratio.

k Others include propylene, ethyl acetate, and crotonaldehyde.

1 Ethyl acetate: 2.4, Butanol: 0.8, Propylene: 2, C_6_^+^: 9.3. ^2^Propylene: 1.8, Pentene: 0.8, C_2_‐C_5_ alkanes: 1.0, Others: 3.3. ^3^Propylene: 2.3, Pentene: 0.7, C_2_‐C_5_ alkanes: 7.7, Others: 2.6.

A recent study explored the catalytic performance of uniformly distributed Zr‐nanoparticles on mesoporous alumina‐carbon [96]. The excellent catalytic activity was due to a synergistic interaction between Zr–Al dual active centers, arising from the confinement effect of mesoporous channels. Acid–base site characterization showed that varying Zr loading modulated the fraction and total active site content, with the 10 wt% Zr/Al–C exhibited the highest total acid and base site density [96]. The superior acid–base profile at 10 wt% Zr loading compared to 5, 20, and 30 wt% Zr was likely due to an optimal Zr–Al^3+^ interaction that promoted high dispersion, prevented sintering, and preserved surface area [96]. Accordingly, the catalyst achieved approximately 58% BD selectivity and almost 95% ethanol conversion. It also demonstrated excellent stability for over 120 h time‐on‐steam at 648 K [97]. In another study, Co modification was reported to suppress the surface acidity of yttrium phosphate‐based catalysts [97]. Co^2+^ species coordinated with PO_4_^3−^ groups, partially suppressing Lewis acid sites while enhancing dehydrogenation and C–C coupling. This modification steered product selectivity toward BD while suppressing dehydration byproducts [97]. However, higher Co loading was detrimental, likely due to excessive coverage of active acid sites [97].

Silica‐supported Ag is well‐established for ethanol dehydrogenation (Table 4). Mamedov et al. thus developed multifunctional catalysts by physically mixing Ag/SiO_2_ with Lewis acid cation‐supported *β*‐zeolites or SiO_2_ and incorporating other elements, such as Ta and Y [98]. The distribution of C_4_ products, such as BD, was directly correlated to the Lewis acid strength [98]. Interestingly, the BD selectivity over 2Ta‐SiO_2_ ranged from a mere 12% at low ethanol conversion (15%) to almost 67% at high conversion (70%), revealing a strong correlation of BD selectivity with ethanol conversion [98]. However, relatively higher BD selectivity was observed over 2Y‐SiO_2_ at low ethanol conversion, stabilizing around 50% at higher ethanol conversions [98]. Moreover, BD selectivity was increased from 12% to 24% by increasing Ta loading on SiO_2_ from 2 to 10 wt%, whereas for the Y catalysts, BD selectivity was practically unaffected by Y loading on SiO_2_ [98]. On the other hand, butanol selectivity was raised correspondingly by an increase in Y loading [98]. The confinement effects of microporous zeolite were found to reduce the size of metal cation clusters formed during doping, leading to enhanced active site isolation with improved coupling reaction and turnover frequency compared to their analogous SiO_2_ supports [98].

Dai et al. enhanced the performance and stability of a bimetallic ZnY/BEA zeolite by introducing Cu metal [99]. The Cu metal forms tetracoordinated Cu(II) species with an optimum acid–base ratio. The trimetallic CuZnY/BEA thus exhibited excellent catalytic performance with 99% ethanol conversion and almost 74% BD selectivity at 623 K [99]. However, a further increase in temperatures led to a decreased BD selectivity due to accelerated aldol condensation, with undesired heavier byproducts [99]. The alkali metal doping was reported to improve BD selectivity by inhibiting undesirable dehydration reactions [100]. Jiang et al. further demonstrated that Ta incorporation on MgO–SiO_2_ can significantly improve ethanol conversion (around 35%–55% increase) and BD selectivity [69]. FTIR analysis confirmed the formation of the Ta–O–Si bond in the doped MgO‐SiO_2_, generating strong Lewis acid sites that facilitated aldol condensation and MPV reactions, thereby boosting BD selectivity [69]. The Ta‐doped catalyst also demonstrated exceptional stability, maintaining 64% ethanol conversion and 80% BD selectivity after 24 h time‐on‐steam [69]. However, the commercial application of Ta‐based catalysts is limited due to its scarcity. The mesoporous bifunctional Cu‐Ta‐SiO_2_ was prepared by acetamide elimination synthesis, and it achieved appropriate Lewis acidity and Cu metal nanoparticle dispersion, yielding a higher BD selectivity (approximately 74%) [101].

### 4.2 Zirconia‐Based Catalysts for the Two‐Step ETB Process

Limited attention has been paid to Zr‐based catalysts for the one‐step process. However, Lewis acidic silica‐supported zirconia, tantalum, and niobium oxides were extensively studied for the two‐step ETB process in the past decade [46, 102]. The redox properties of ZrO_2_ catalyze the MPV reduction of 3‐hydroxybutanal [103]. In one of the earlier studies, Jones et al. investigated a ternary mixture comprising ZrO_2_, ZnO, and silica [104]. The ZnO facilitated ethanol dehydrogenation, while the combination of silica and Zr species promoted acetaldehyde aldol condensation, and the crotonaldehyde reduction by ethanol [104]. The resulting crotyl alcohol readily dehydrated to BD [105]. The BD selectivity was improved at higher temperatures due to faster acetaldehyde consumption. Several subsequent studies have since highlighted pure and modified zirconium as viable catalysts (Table 5).

**TABLE 5:** Catalytic efficacy of zirconia‐based catalysts in the two‐step conversion of ethanol to 1,3‐butadiene at 1 bar.

SN	Catalyst	**WHSV** (h^−1^)	Et/AA ratio	**T** (K)	**N**_**2**_ (ml/min)	X (%)	Selectivity (%)						References
							BD	Ac	E	DEE	B	Others	
1^a^	1.5% Zr‐0.5% Zn/SiO_2_ (60)^b^	0.75^d^	—	648	25^e^	60.9	34.8	4.4	52.2	4.5	4.2	—	[105]
	1.5% Zr‐0.5% Zn/SiO_2_ (60)^c^	1.5^d^				46.0	38.9	10.3	41.1	6.7	3.0	—	
	1.5% Zr‐0.5% Zn/SiO_2_ (150)^c^	1.5^d^				48.0	47.9	9.4	25.8	14.0	3.0	—	
	1.5% Zr‐0.5% Zn/SiO_2_ (60)^b^	1.5^d^		673		50.0	45.8	13.8	30.7	6.0	3.7	—	
2	ZrO_2_ f	Et^g^	—	573	N	6.1	—	11	89		—	—	[65]
	4% ZrO_2_/SiO_2_ f					10	—	10	90		—	—	
	1% Ag‐4% ZrO_2_/SiO_2_ f					20	36	47	15		—	2	
	4% Ag‐4% ZrO_2_/SiO_2_ f	0.23		598		91.6	68.3	—	8.8	—	6.4	15.3^h^	[96]
3	2% ZrO_2_/SiO_2_	1.8^i^	1.5:1	623	50	~40	~55	—	~7.5	~23	~14	—	[106]
	5.6% ZrO_2_/SiO_2_					—	~35	—	~7.5	~25	~18	—	
	8.4% ZrO_2_/SiO_2_					—	~37	—	~7.5	~22.5	~22.5	—	
	2% ZrO_2_/SiO_2_	1.8^i^				~40	~20	—	—	—	~58	—	
		1.8^i^	3.5:1			~42	~70	—	—	—	<1	—	
4	ZrO_2_/SiO_2_	1.8	3.5:1	593	40	5.33	64.09	—	0.34	8.42	—	29.28^1^	[67]
	ZrO_2_/nano‐SiO_2_					52.39	91.43	—	2.26	3	—	3.3^2^	
5	0.5% ZnO/ZrO_2_–SiO_2_–Al_2_O_3_	1.8	3.5:1	593	—	36.8	83.5	—	5.1	3.7	0.4	7.5^3^	[107]
	1% MgO/ZrO_2_–SiO_2_–Al_2_O_3_					27.0	75.3	—	11.1	8.1	0.7	4.9^4^	

X, Conversion; Et, Ethanol; E, Ethylene; DEE, Diethyl ether; Ac, Acetaldehyde, and B, Butylene. All metal loadings are in wt%. N, Fixed amount of N_2_ as carrier gas.

a Number in () represent pore diameter of the silica in °A.

b Calcination temperatures: 573 and 773 K, respectively.

c Calcination temperatures: 573 and 773 K, respectively.

d LHSV.

e He carrier gas.

f Silica source: Davisil 636 and Davisil 646, respectively.

g Feed stream consists of 6 kPa ethanol balanced by N_2_.

h Others include propylene, butanol, crotonaldehyde, and unidentified hydrocarbons.

i Unit is g_ethanol_ g_cat_^−1^ h^−1^.

1 Ethyl acetate: 3.04, Butanol: 14.62, Others: 11.26. ^2^Ethyl acetate: 0.51, Butanol: 0.71, Propylene: 0.48, Others: 1.60. ^3^Ethyl acetate: 1.1, Butanol: 0.2, Propylene: 1.4, C_6_^+^: 4.8. ^4^Ethyl acetate: 0.8, Propylene: 1.5, C_6_^+^: 2.6.

Miyake et al. compared the acid–base properties of Ag‐promoted ZrO_2_–SiO_2_ with those of bulk ZrO_2_ [64]. Their study revealed that the basic sites of bulk ZrO_2_ were associated with surface oxygen anions, which were stronger than those of silica‐supported ZrO_2_. The reaction results demonstrated that Ag‐supported ZrO_2_ dispersed on silica outperformed bulk ZrO_2_ under identical conditions [64]. Han et al. thus examined the performance of a series of ZrO_2_–SiO_2_ catalysts synthesized via the sol–gel method [108]. Their study highlighted the impact of ZrO_2_ loading on the acid–base balance, a crucial factor influencing BD yield. The ZrO_2_ content played a critical role in catalytic performance due to the well‐known redox properties of Zr. The ZrO_2_ loading of 2 wt% exhibited nearly 70% BD selectivity under optimum reaction conditions [108]. Further investigation revealed that the sol–gel synthesis facilitated the formation of relatively weak Lewis acid sites and effective dispersion of ZrO_2_ on amorphous silica, which were the key factors contributing to the strong catalytic performance [108]. The effect of ZrO_2_ dispersion on catalytic activity was further verified by Gao et al., and they achieved approximately 91% BD selectivity by incorporating ZrO_2_ onto nano‐SiO_2_ with a high specific surface area [66]. The acidity of ZrO_2_–SiO_2_ was also another critical factor to facilitate the transformation of acetaldehyde into BD precursors [106]. It was observed that increasing ZrO_2_ loading beyond the optimal level resulted in extreme acidity, with a negative effect on BD selectivity and excessive formation of butylene due to reduced dehydrogenation activity. These findings highlight that achieving high BD selectivity requires a balance between dehydrogenation and dehydration sites [108].

The modification of ZrO_2_–SiO_2_ with Ag metals enhanced the catalytic performance in the ETB reaction [64]. The introduction of Ag improved ethanol dehydrogenation, while ZrO_2_ retained its activity for C–C coupling and dehydration reactions. By balancing between Ag and ZrO_2_ active sites, they achieved 65% BD selectivity and 75% ethanol conversion [64]. Similarly, the incorporation of ZnO and alkali metals into the ZrO_2_–SiO_2_ was reported to improve BD selectivity by inhibiting undesirable dehydration reactions [109]. Moreover, silica‐supported zirconium oxides were observed to yield higher amounts of C_6_^+^ hydrocarbons, likely due to their tendency to catalyze condensation reactions [34]. In recent times, Y‐doped dealuminated *β*‐zeolites were investigated for ETB conversion [107]. The active site quantification on various Y‐doped dealuminated *β*‐zeolites was performed via in situ and ex situ pyridine titration, as well as kinetic analysis to enable further developments, including multimetallic catalysts [107].

### 4.3 Emerging Catalyst Formulations

**FIGURE 6:**
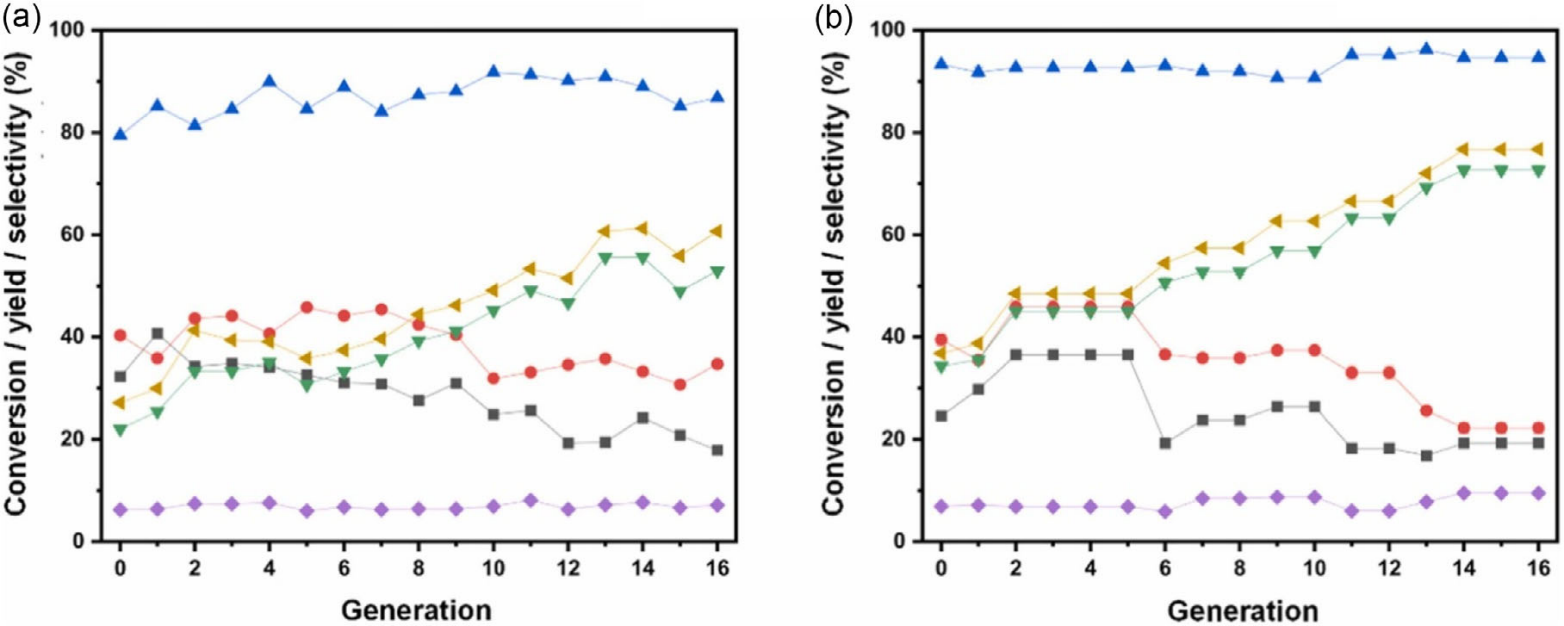
Evolution of the catalysts using genetic algorithm. (a) The average performance of 20 catalysts in each generation, and (b) the performance of the best catalyst in each generation, excluding the elites, are plotted against the generation. (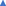): EtOH conversion; (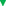): BD yield; (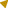): BD selectivity; (▪): ethylene yield; (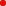): AcH yield; (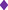): butene yield. Reproduced with permission [111].

There is a growing trend of leveraging combinatorial approaches and data‐driven catalyst screening using machine learning (ML) and high‐throughput experimentation (HTE). Jayakumar et al. applied a genetic algorithm (GA)‐driven HTE strategy to explore 14 metals cosupported on mesoporous silica [110]. The ML was further employed to analyze the GA‐derived dataset and extract mechanistic insights into catalyst performance. The GA approach identified several highly effective catalysts, primarily comprising Mg, Zn, Y, and Hf, with secondary components, such as Zr, Nb, and La [110]. The best‐performing catalyst achieved approximately 90% ethanol conversion and around 77% BD selectivity (Figure 6) [110]. The integration of XGBoost regression and Shapley's feature importance analysis further provided valuable insights into the roles of each element [110]. However, such data‐driven approaches are scarce in the literature. The screening of supports is also important to overcome low BD selectivity and poor catalyst stability. Zhao et al. recently introduced a dendrite‐structured mesoporous silica (DSMS) support to develop a highly robust Zn–Zr catalyst, achieving atomic‐level dispersion of active sites [111]. The Zn–Zr/DSMS exhibited remarkable catalytic performance and exceptional stability (Table 6) [111]. The unique dendritic structure played a pivotal role in preserving catalytic activity, thereby reinforcing the critical influence of support structure [111]. Further research should prioritize the rational design of supports for uniform active site dispersion and enhanced stability under industrial conditions.

**TABLE 6:** Catalytic efficacy of emerging catalyst formulations in the conversion of ethanol to 1,3‐butadiene at 1 bar.

SN	Catalyst	**WHSV** (h^−1^)	Et/AA ratio	**T** (K)	**N**_**2**_ (ml/min)	X (%)	Selectivity (%)						References
							BD	Ac	E	DEE	B	Others	
1^a^	Mg: 9; Zn: 16; Cu: 7; Ag: 4; Ni: 8; Al: 2; La: 0; Y: 25; Hf: 24; Zr: 0; Cr: 1; Ga: 2; Nb: 1; Mo: 1.^b^	18.3^c^	—	673	200^d^	95	73	22	19	—	9	—	[111]
	Mg: 9; Zn: 12; Cu: 7; Ag: 4; Ni: 8; Al: 2; La: 0; Y: 25; Hf: 24; Zr: 0; Cr: 1; Ga: 2; Nb: 5; Mo: 1.^b^					94	67	30	19	—	8	—	
	Mg: 9; Zn: 15; Cu: 8; Ag: 5; Ni: 8; Al: 2; La: 7; Y: 10; Hf: 18; Zr: 9; Cr: 1; Ga: 2; Nb: 5; Mo: 1.^b^					96	69	26	17	—	8	—	
	Mg: 26; Zn: 15; Cu: 7; Ag: 4; Ni: 0; Al: 2; La: 7; Y: 14; Hf: 7; Zr: 14; Cr: 1; Ga: 2; Nb: 1; Mo: 0.^b^					93	68	32	15	—	6	—	
2	0.5% Zn‐2% Zr/DSMS	0.96	—	623	40	70.9	63.9	17.1	5.1	2.4	—	14.4	[112]
	0.5% Zn‐2% Zr/SiO_2_ (meso)					44.1	37.1	33.5	12.2	7.9	—	10.4	
	0.5% Zn‐2% Zr/Silicate‐1					55.9	7.9	48.7	19.6	15.8	—	10.7	
	0.5% Zn‐2% Zr/SBA‐15					40.8	38.6	44.3	5,6	2.5	—	10.8	

X, Conversion; Et, Ethanol; E, Ethylene; DEE, Diethyl ether; Ac, Acetaldehyde, and B, Butylene.

a Catalyst composition.

b Total loading: 1.05 mmol/g‐support.

c Ethanol flow rate is 18.3/35.6 ml/min.

d Ar is used as carrier gas.

### 4.4 Effect of Process Parameters

#### 4.4.1 Effect of Temperature and Weight‐Hourly Space Velocity

Temperature and weight‐hourly space velocity (WHSV) are critical parameters. Da Ros et al. applied a statistical experiment design approach to model catalytic behavior of K_2_O–ZrO_2_–ZnO/MgO–SiO_2_ as a function of temperature and WHSV [112]. This model was further validated by González et al. for the Hf–Zn catalyst [39]. The observed trends aligned well with the literature [112]. The high reaction temperatures and low WHSV favored ethanol conversion [72]. This trend has also been widely reported in the literature for the one‐step [80, 109, 113, 114] and two‐step [115] processes, consistent with the endergonic nature of the reaction. However, ethanol conversion over ZnO/Al_2_O_3_ declined between 673 and 723 K, possibly due to thermodynamically unfavorable aldol condensation reaction at higher temperatures [116].

**FIGURE 7:**
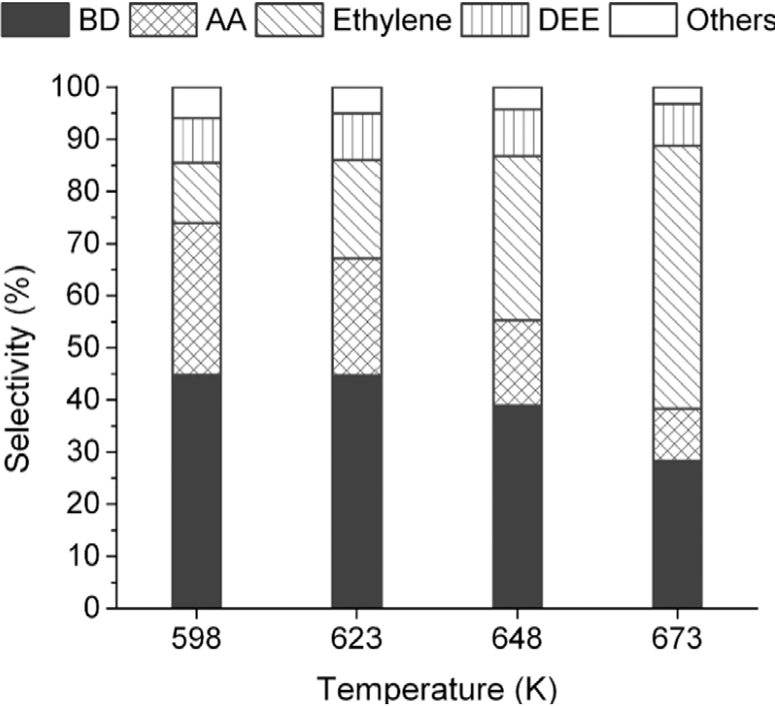
Effect of temperature on product selectivity over Cu‐Ta/SiBEA catalyst (WHSV = 1.5 g_ethanol_ g_cat_^−1^ h^−1^). Reproduced with permission [117].

**FIGURE 8:**
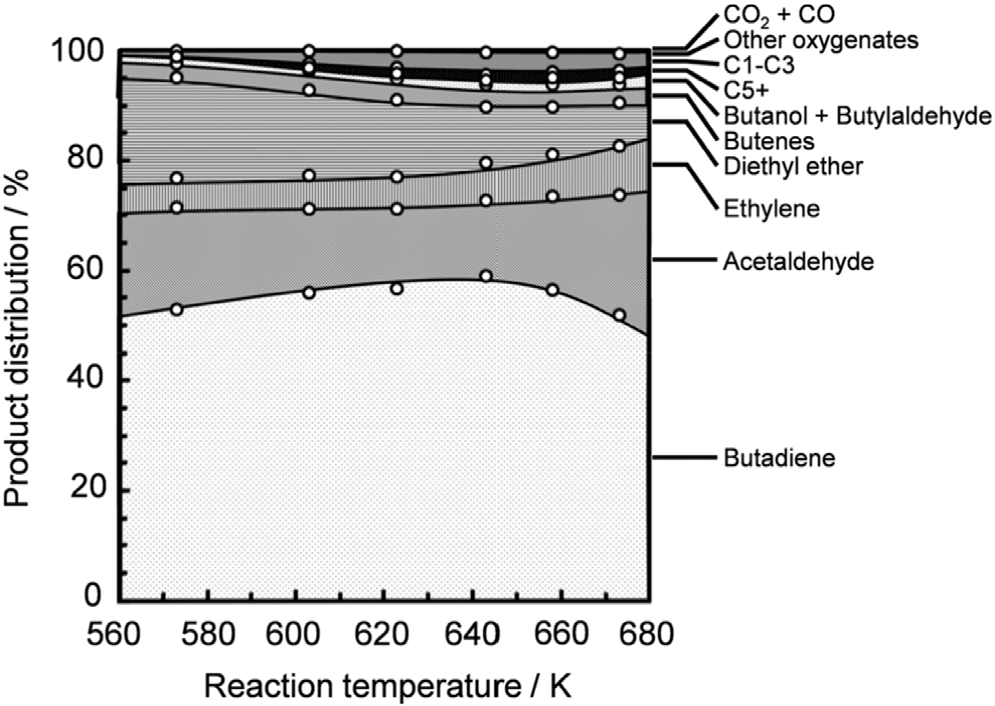
Effect of temperature on product selectivity over Zn/talc catalysts (WHSV = 1.76 × 10^−4^ mol g^−1^). Reproduced with permission [119].

The BD selectivity was decreased at elevated WHSV, with a corresponding rise in acetaldehyde [112]. These results align well with the mechanism, where aldol condensation is the rate‐limiting step with the accumulation of acetaldehyde at lower WHSV [27, 34]. In contrast, BD selectivity was enhanced by increasing reaction temperatures, with the simultaneous decline in acetaldehyde selectivity [79, 112]. However, wide variations in BD selectivity with temperature have been reported for diverse catalysts. Kyriienko et al. observed a significant decline in BD selectivity at elevated temperatures above 573 K, with ethylene becoming the dominant product for the one‐step process over Cu‐Ta/SiBEA (Figure 7) [118]. This trend was also observed for 3%Au/50%MgO–SiO_2_, where BD selectivity initially showed improvement with rising temperatures, and then decreased above 573 K [71]. Interestingly, BD selectivity remained relatively constant across the temperature range over MgO–SiO_2_, when ethanol conversion was maintained at 40% by adjusting WHSV (Figure 8) [117]. However, the distribution of byproducts, including acetaldehyde, ethylene, DEE, etc., was noticeably affected under these conditions [117]. These inconsistent trends are difficult to generalize and are largely due to the considerable variations in catalyst composition and reactor configurations in different studies. In general, within the optimal temperature range of 602–703 K, higher temperatures favored ethanol conversion [10]. However, the product selectivity was dependent on the nature of the catalyst [10]. Moreover, lowering WHSV enhances BD selectivity. However, this improvement often comes at the expense of low overall BD productivity [112]. Conversely, increasing WHSV enhances BD productivity, though only up to a certain threshold, beyond which BD formation is suppressed, likely due to kinetic or mass transport limitations.

#### 4.4.2 Effect of Pressure

**FIGURE 9:**
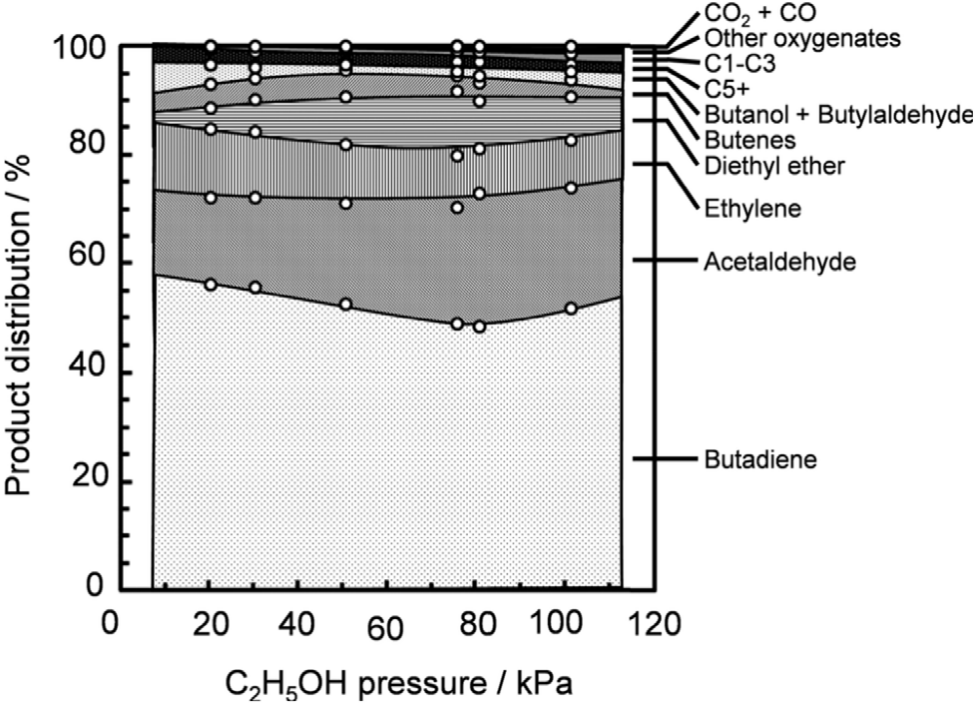
Effect of ethanol pressure on product selectivity over Zn/talc catalyst (WHSV = 1.76 × 10^−4^ mol g^−1^). Reproduced with permission [119].

Makshina et al. demonstrated that the equilibrium ethanol conversion was higher at low pressures due to the negative change in the number of moles in the reaction [10]. The loss of ethanol conversion at higher partial pressures can be compensated by elevating reaction temperature, which comes at the cost of enhanced byproduct formation. However, Hayashi et al. observed nearly constant (~42%) ethanol conversion over Zn/talc at 673 K and 5.49 g h mol^−1^ over 20.3–101.3 kPa ethanol pressures (Figure 9) [119]. The BD formation was increased proportionally with ethanol pressure, indicating a first‐order correlation between BD formation and feed pressure [117]. However, BD selectivity was decreased slightly with increasing ethanol pressure, with a gradual increase in acetaldehyde [117]. The decreasing trend of BD selectivity with pressure was further verified by Kvisle et al., which agreed with thermodynamic calculations, considering a positive change in the number of moles in the reaction [85]. Furthermore, lighter olefins, such as ethylene, were decreased, whereas butylene selectivity was only slightly affected by ethanol pressure [117].

#### 4.4.3 Effect of Water in the Feed

The use of aqueous ethanol is an important strategy to save energy for concentrating ethanol from fermentation broth, which forms an azeotrope at around 93 wt%. Water is also formed during the reaction, which may alter the reaction equilibrium [39]. Three major effects of water were observed in the one‐step process: (i) suppressing ethanol conversion, (ii) altering product selectivity, and (iii) reducing coke formation [39, 56, 119]. The reduction in ethanol conversion was due to the inhibition of ethanol dehydrogenation sites by competitive adsorption of ethanol and water on Lewis acid–base pairs of Hf–Zn [39]. However, the relationship between ethanol conversion and water content is not linear. A lower reduction in ethanol conversion was observed at high temperatures that counteracts the effect of water [39]. The acetaldehyde yield was increased with water content [39]. It was due to the greater inhibition of aldol condensation compared to ethanol dehydrogenation, resulting in a decrease in the BD yield and other heavier products [39]. The IR spectra indicated that Brønsted acid sites were evolved in MgO–SiO_2_ by interaction of water with Lewis acidic sites [119]. However, the high SiO_2_ content was detrimental to BD selectivity owing to the combination of the above effect and segregation of Si, as the high acidity favored ethanol dehydration [119]. However, the variation of BD selectivity due to the water can be tuned by altering the reaction conditions [13]. The lower catalyst deactivation was attributed to the adsorption of water on Lewis acid sites, preventing the formation of heavier carbonaceous species by acetaldehyde condensation [116, 119]. Similar effects were also observed for the two‐step process, where heavier carbonaceous products were suppressed in the presence of water. Zhang et al. and Zhu et al. found that the ethanol–acetaldehyde conversion was slightly reduced, and BD selectivity was practically unaffected over ZnO–ZrO_2_ and MgO–SiO_2_ up to 50 wt% water in the feed [115, 120].

#### 4.4.4 Effect of Ethanol/Acetaldehyde Ratio

**FIGURE 10:**
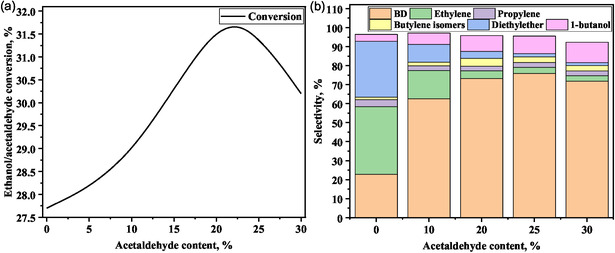
Effect of acetaldehyde content on (a) ethanol/acetaldehyde conversion and (b) product selectivity over MgO‐SiO_2_ catalyst. **Reaction conditions**: 623 K and acetaldehyde content, wt% = 100 × mass of acetaldehyde/(mass of ethanol+acetaldehyde) [116].

The ethanol/acetaldehyde ratio plays a critical role in BD yield for the two‐step process, with limited studies available in the literature [108]. The addition of acetaldehyde to the feed greatly improved the catalytic activity of MgO–SiO_2_ and BD selectivity [115]. Interestingly, the catalytic activity and BD selectivity displayed a volcanic trend with acetaldehyde content in the feed (Figure 10) [115]. Increasing the acetaldehyde content over an optimum showed increased butanol selectivity, whereas higher ethanol content in the feed was favorable for ethylene and DEE formation [115]. However, the optimal value differs across different catalysts, ostensibly due to the differences in their properties and the reaction conditions [115, 121]. Therefore, the ethanol/acetaldehyde ratio must be studied further to enhance BD selectivity in the two‐step process.

### 4.5 Catalyst Deactivation

Catalyst deactivation remains a critical challenge in catalytic processes, limiting the scope of their commercial application. Although catalyst deactivation is often unavoidable, its impact can sometimes be mitigated by delaying/minimizing deactivation or reversing the activity loss. Consequently, understanding catalyst deactivation is vital for the research, development, and optimization of operating conditions. An understanding of the deactivation mechanism is crucial for designing robust catalysts. However, the catalyst stability study and deactivation mechanisms remain relatively underexplored. This section thus provides a comprehensive review of existing studies on catalyst deactivation, causes of deactivation, the susceptibility of different catalysts, and strategies to mitigate these effects.

#### 4.5.1 Causes of Catalyst Deactivation

##### 4.5.1.1 Coking

Strong acidic and basic sites are believed to accelerate the formation of carbonaceous compounds [113, 122]. Coke formation is identified as one of the primary reasons behind catalyst deactivation. These carbonaceous deposits block active sites and restrict the access of the reactants, reducing the overall catalytic activity. Yan et al. elucidated the catalyst deactivation mechanism for Zn‐Y/*β*, employing several catalyst characterization methods, such as MS, in situ DRIFTS, UV–vis, TGA, etc [54]. The large unsaturated aldehydes/ketones were deposited over catalytically active sites, leading to the gradual deactivation [54]. Other groups also reported similar observations [101, 123, 124].

##### 4.5.1.2 Active Site Poisoning

Active site poisoning involves chemical bond formation between active sites and products/intermediates. González et al. investigated the catalyst deactivation of the Hf–Zn [39]. They identified the retention of aromatic‐type carbonaceous species over dehydrogenating Zn^2+^ sites as the leading cause of deactivation. Product retention led to the conversion of reduced Zn^2+^ active sites to ZnO, resulting in a misbalance between Hf^4+^ and Zn^2+^, significantly affecting the catalyst performance [39].

##### 4.5.1.3 Structural Changes

Taifan et al. investigated the deactivation of CuO‐ and ZnO‐modified MgO–SiO_2_ [90]. The operando X‐ray analysis of the Cu‐modified catalyst at 673 K revealed that the Cu–O bonds broke down over time, simultaneously forming Cu–Cu pairs. It was attributed to Cu reduction and particle sintering, with a decrease in catalytic activity [90]. In contrast, the Zn‐modification showed stable zinc bonds under the same conditions. However, coke deposition was identified as the primary cause of deactivation for the ZnO‐containing catalysts [90].

#### 4.5.2 Strategies to Control Catalyst Deactivation

Several strategies have recently been explored to mitigate catalyst deactivation. Incorporating dopants like Zn has been shown to balance acidity and basicity, thereby reducing undesired side reactions responsible for coke formation [125]. Moreover, hierarchical or trimodal catalyst improves the diffusion of reactants/products and minimizes the accumulation of carbonaceous species [61, 126]. The cofeeding of water has emerged as another effective strategy [39]. While water has been observed to poison Lewis acid sites, it can simultaneously generate new Brønsted acid sites [39]. It reduces the formation of coke and prolongs the operational lifespan of the catalysts. Passivating acid sites is another strategy to inhibit the over‐condensation of carboxylic intermediates and improve catalytic stability [100]. The alkali‐doping over silica‐supported Ag/ZrO_2_ decreased the Lewis acid sites, and Ag particles were advantageous in preventing undesirable ethanol dehydration reaction and formation of heavier aldol condensation products [100]. Taifan et al. observed the sintering under reaction conditions, while González et al. found the reduction of metal oxides during the reaction, which hindered catalytic activity [39, 90]. To mitigate current challenges, the catalyst should be designed with optimized textural and acid–base properties for enhanced coke resistance. The research should also focus on regeneration techniques, such as oxidative or reductive treatments, to restore active sites.

## 5 Techno‐Economic, Life Cycle Analysis (LCA), and Pilot‐Plant Study

Techno‐economic analysis (TEA) and LCA are crucial in determining the economic viability and environmental sustainability of the process. In a recent study, Camacho et al. compared the economic and environmental sustainability of the one‐step and two‐step processes using experimental results of highly selective catalysts tested under simulated industrial conditions for a rigorous assessment [127]. These results were compared with the conventional naphtha‐based BD production route. The analysis considered sugarcane‐based ethanol under Brazilian conditions and incorporated stochastic simulations to account for variability in feedstock cost, utility pricing, and other process inputs. Their findings identified ethanol price as the dominant factor in overall economics. The two‐step process exhibited higher BD selectivity, with 10%–35% lesser ethanol consumption compared to the one‐step technology. However, the higher BD selectivity was obtained by operating with large ethanol recycles in the second stage that significantly increased the specific energy consumption (52–59 GJ/t BD), almost twice that of the one‐step process (25–29 GJ/t BD). The two‐step process thus demonstrated a higher production cost (2380–2427 €/t) than the one‐step process (2006–2247 €/t). However, ETB processes showed a significant reduction in CO_2_ emissions (7.6%–103%) compared to naphtha‐derived BD, with the one‐step process being more favorable [127].

In a separate study, the same group assessed the economic feasibility of 200 ktons/year BD production facility [16]. Assuming an average ethanol price of €450 per cubic meter, the minimum BD selling price (MBSP) required to achieve a 10% return on investment was found to be 1.13 and 1.26 times the current BD market price (€1529/ton) [16]. While the ETB technology can be economically viable under favorable ethanol and BD price conditions, its profitability largely depends on improving catalyst selectivity. More efficient catalysts would help lower the amount of ethanol needed per ton of BD, which is critical given that ethanol accounts for dominant production costs [16]. The LCA revealed that the ETB process reduced GHG emission by 8%–26% in comparison to fossil‐derived BD production [16]. However, this environmental benefit comes at the cost of significantly higher water use (62–137 times) and greater energy demand (50–250% increase) [16]. Farzad et al. examined the integration of the ETB process with a sugar mill in Brazil [128]. The economic evaluation indicated a nonprofitable scenario with the MBSP between USD 2385‐2645/t, which was higher than the current BD market price of USD 800/t (5‐year (2007–2011) average), even though it showed almost 85% reduction in GHG emissions [128]. Nevertheless, process integration helped reduce infrastructure costs and improved overall viability [128]. Notably, their simulations were based on experimental data from older catalyst systems [128]. A deeper understanding of the mechanism can generate more realistic input data for process simulations, with a reliable prediction. As of now, no dedicated TEA of the Lebedev process has been reported, likely due to insufficient kinetic data.

In early 2024, Michelin, in collaboration with IFPEN and Axens, commissioned the first industrial‐scale demonstration for bio‐based BD production using a Lebedev‐type process [129]. This milestone represents a significant advancement in commercializing renewable BD technologies. The successful scale‐up and commercialization of ETB processes require several strategic interventions, including integration with existing petrochemical complexes, the use of renewable energy sources, and the development of selective catalysts [127]. Moreover, flexible, multiproduct biorefinery designs can enhance process resilience by buffering against market fluctuations in feedstock and product prices [130, 131]. This modularity in processing allows the facility to dynamically adjust its product portfolio. For example, if global fuel prices rise, making biofuels more feasible, the biorefinery can prioritize fuel production to capitalize on the higher margins. On the other hand, if fuel prices fall and demand shifts toward renewable chemicals or green solvents, the facility can redirect its fermentation and downstream processing systems toward producing these value‐added products. This operational flexibility not only maximizes economic return but also buffers the biorefinery against the volatility of commodity markets. By avoiding dependence on a single product line, the plant remains economically viable and competitive even under changing market conditions to meet with an essential feature for long‐term sustainability in the bioeconomy.

The ethanol contributes the largest operating costs in the ETB process, with a significant influence on MBSP [127]. Therefore, reducing ethanol cost is critical for enhancing the overall economic feasibility. The sugarcane and sugar beet are favorable feedstocks for bioethanol production compared to corn, owing to the fewer processing steps [132]. Conversely, starchy feedstocks provide the highest ethanol yield [132]. On the other hand, cellulosic ethanol was reported to be 18% cheaper than corn ethanol [132]. The projected production cost was approximately USD 495 per cum of ethanol from inedible lignocellulosic biomass, which was only about 8% higher than gasoline costs [132]. Therefore, future research efforts should focus on developing (i) low‐cost ethanol production technology, (ii) an energy‐integrated process with reduced utility consumption, and (iii) BD‐selective catalysts. These developments will eventually make bio‐based BD economically competitive.

## 6 Summary and Outlook

Ethanol‐to‐1,3‐butadiene (ETB) processes have garnered immense attention due to the global push toward sustainable and environmentally benign 1,3‐butadiene (BD) manufacturing through the circular economy. The present review highlights the progress in mechanistic insights and multifunctional catalysts. Although the exact mechanism remains debatable, a widely accepted pathway involves ethanol dehydrogenation, followed by acetaldehyde self‐aldol condensation and MPVO‐dehydration of crotonaldehyde to BD. Techniques, such as Isotope labeling, in situ DRIFTS studies, and DFT modeling, have further advanced the understanding of intermediate species involved in the reaction mechanism. Conversely, thermodynamic analysis validates the feasibility of the proposed mechanisms. MgO–SiO_2_‐based catalysts demonstrate high catalytic activity in the one‐step process. However, their performance is sensitive to acid–base site balance. Introducing dehydrogenating metals (Ag and Zn) enhances acetaldehyde formation. Alkali doping moderates acid strength, reducing side reactions and coke formation. Rare‐earth modification, such as yttrium phosphate‐based composites, further improves BD selectivity and catalyst stability. ZrO_2_‐based catalysts are preferred in the two‐step process due to their tunable acidity and mechanical robustness. However, coke deposition and metal sintering issues persist. However, process economics are adversely affected by high ethanol costs and energy consumption. Co‐locating ETB plants with bioethanol facilities lowers infrastructure and operational costs. On the other hand, LCA emphasizes the need for trade‐offs between reducing GHG emissions and increased water usage. The future research should focus on (i) translating mechanistic insights from in situ and DFT studies into catalyst design with optimum acid–base pairs and metal sites, (ii) catalyst regeneration protocols to address deactivation, (iii) use of low‐cost feedstock for bioethanol production to improve process economics, and (iv) process integration to reduce energy costs. Overall, the combination of advanced catalyst characterization, in situ studies, thermodynamic analyses, DFT calculations, kinetic modeling, and sustainability evaluation is the key to the successful development of commercially viable ETB technologies.

## Nomenclature

BD: 1,3‐butadiene
DEE: diethyl ether
DFT: density functional theory
DRIFTS: Diffuse Reflectance Infrared Fourier Transform Spectroscopy
ETB: ethanol‐to‐BD
GA: genetic algorithm
LCA: life cycle analysis
MBSP: minimum BD selling price
ML: machine learning
MPV: Meerwein–Ponndorf–Verley
MPVO: Meerwein‐Ponndorf‐Verley‐Oppenauer
TEA: techno‐economic analysis
WHSV: wight‐hourly space velocity

## Supporting Information

Additional supporting information can be found online in the Supporting Information section. **Supporting** **Fig. S1:** Dual‐cycle mechanism for the conversion of ethanol into 1,3‐butadiene. **Supporting** **Fig. S2:** Reduction of crotonaldehyde to 1,3‐butadiene using dissociated hydrogen over MgO catalyst. **Supportin** **g** **Table S1:** Reaction network and kinetic model of the one‐step ETB reaction over (K_2_O)ZnO/γ‐Al_2_O_3_ catalyst in the presence of H_2_O_2_ initiator. **Supporting** **Table S2:** Global kinetic reaction scheme, pre‐exponential factors, and activation energies of the model developed by Dussol et al. for two‐step ETB reaction over Ta_2_O_5_–SiO_2_ catalyst. **Supporting** **Table S3:** Standard Δ*H* and Δ*G*of reactions involved in proposed mechanisms.

## Conflicts of Interest

The authors declare no conflicts of interest.

## Supporting information

Supplementary Material
