# Supplementary material for: Recent Advances in Catalytic Conversion of Bioethanol to 1,3‐Butadiene: Reaction Mechanism, Catalyst Design, and Process Scalability
Source: ChemSusChem. 2025 Nov 26;19(1):e202501926. doi: 10.1002/cssc.202501926 (PMC12767281; doi:10.1002/cssc.202501926)
Supplement: Supplementary file 1 — Supplementary Material [file CSSC-19-e202501926-s001.pdf]

Supplementary Information

Recent Advances in Catalytic Conversion of Bioethanol to 1,3-Butadiene: Reaction Mechanism, Catalyst Design, and Process Scalability

Abhishek R. Varma [1], Md Ziyaur Rahman [1,2], Siddharth Gadkari [3], Atthasit Tawai [4], Malinee Sriariyanun [4], Ao Xia [5], Vinod Kumar [6,7,*], Sunil K. Maity [1,*]

[1] *Department of Chemical Engineering, Indian Institute of Technology Hyderabad, Kandi, Sangareddy-502284, Telangana, India*

[2] *Presently working: Advanced centre for energetic material (ACEM), DRDO, Ambe Hill, Ozhar, Nasik-422007, Maharashtra, India*

[3] *School of Chemistry and Chemical Engineering, University of Surrey, Guildford GU2 7XH, UK*

[4] *The Sirindhorn International Thai-German Graduate School of Engineering, King Mongkut's University of Technology North Bangkok, Bangkok 10800, Thailand*

[5] *Institute of Engineering Thermophysics, School of Energy and Power Engineering, Chongqing University, Chongqing 400044, China*

[6] *Magan Centre for Applied Mycology, Faculty of Engineering and Applied Sciences, Cranfield University, Cranfield MK43 0AL, United Kingdom*

[7] *Centre for Sustainable Rural Development, Indian Institute of Technology Roorkee, Roorkee, Uttarakhand, India*

***Corresponding authors:**

1. *Dr. Vinod Kumar, Ph: +44 (0) 1234754786, E-mail: vinod.kumar@cranfield.ac.uk*
2. *Prof. Sunil K. Maity, Ph: +91-40-23016202, E-mail: sunil_maity@che.iith.ac.in*

Table S1
Reaction network and kinetic model of the one-step ETB reaction over $(K_2O)ZnO/\gamma\text{-}Al_2O_3$ catalyst in the presence of H_2O_2 initiator.[1]

#	Reaction Scheme	Rate law of the limiting steps, s^{-1}	Rate constant, s^{-1}
R1	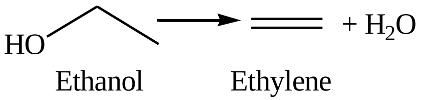	$r1 = \frac{k_1 \cdot X_{C_2H_5OH}}{1+K_A \cdot K_{CH_3CHO}}$	$k_1 = 4.86 \pm 1.2 \times 10^{16} \cdot e^{\frac{-210600 \pm 2100}{RT}}$
R2	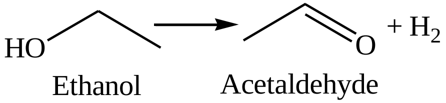	$r2 = \frac{k_3 \cdot X_{C_2H_5OH}}{1+K_B \cdot K_{CH_3CHO} \cdot X_{C_4H_8O}{}^{-1}}$	$k_3 = 2.30 \pm 0.7 \times 10^3 \cdot e^{\frac{-19050 \pm 190}{RT}}$
R3	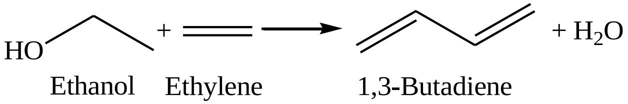	$r3 = \frac{k_5 \cdot X_{C_2H_4}}{1+K_B \cdot K_{CH_3CHO} \cdot X_{C_4H_8O}{}^{-1}}$	$k_5 = 1.26 \pm 0.3 \times 10^2 \cdot e^{\frac{-13650 \pm 136}{RT}}$
R4	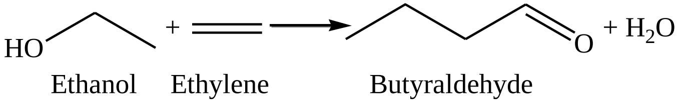	$r4 = \frac{k_8}{1+K_C \cdot X_{CH_3CHO}}$	$k_8 = 2.29 \pm 0.7 \times 10^2 \cdot e^{\frac{-13070 \pm 130}{RT}}$
R5	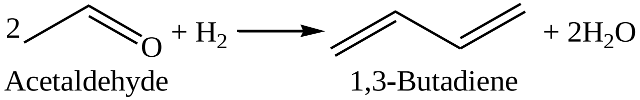	$r5 = \frac{k_{12}}{1+K_B \cdot K_{CH_3CHO} \cdot X_{C_4H_8O}{}^{-1}}$	$k_{12} = 3.76 \pm 1.0 \times 10^3 \cdot e^{\frac{-6890 \pm 70}{RT}}$
R6	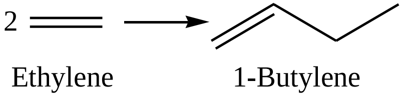	$r6 = \frac{k_{13} \cdot X_{C_2H_4}{}^2}{1+K_A \cdot K_{CH_3CHO}}$	$k_{13} = 3.06 \pm 1.3 \times 10^2 \cdot e^{\frac{-10650 \pm 120}{RT}}$
R7	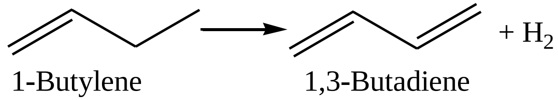	$r7 = \frac{k_{15} \cdot X_{C_4H_8}}{1+K_A \cdot K_{CH_3CHO}}$	$k_{15} = 2.90 \pm 1.0 \times 10^{38} \cdot e^{\frac{-497500 \pm 4900}{RT}}$

X_i refers to the output molar fraction of compound i, K_n represents the equilibrium adsorption constant for active sites A, B, or C, 500, 100, and 550, respectively, The unit of activation energy is J mol^{-1}, The rate law of limiting steps have been modelled considering first-order kinetics.

Table S2

Global kinetic reaction scheme, pre-exponential factors, and activation energies of the model developed by Dussol et al. for two-step ETB reaction over Ta_2O_5–SiO_2 catalyst.[2]

#	Reaction Scheme	Reaction Type	$k^0 \times 10^3$	E_a (kJ mol^{-1})
R1	2Ac ⇌ Crotonaldehyde + H_2O	Aldol reaction	0.3[b]	40
R2	Ac ⇌ MVK + H_2O	Aldol reaction + intra-MPV	6.4×10^{-2}[b]	40
R3	Crotonaldehyde + Et ⇌ 2B1OL + Ac<br>MVK + Et ⇌ 3B2OL + Ac<br>Butanal + Et ⇌ 1-Butanol + Ac<br>MEK + Et ⇌ 2-Butanol + Ac	MPV reaction	36[b]	45
R4	2-Buten-1-ol → BD + H_2O<br>3-Buten-2-ol → BD + H_2O<br>1-Butanol → Butene + H_2O<br>2-Butanol → Butene + H_2O	Dehydration	9.4[a]	55
R5	2-Buten-1-ol ⇌ Butanal<br>3-Buten-2-ol ⇌ MEK	Isomerisation	0.14[a]	
R6	2 Ac + Et ⇌ EtOAc + H_2O + Et	Ester formation	3.6×10^{-4}[b]	41
R7	2Ac ⇌ Acetone + Formaldehyde	Aldol addition + intra-MPV + retro-aldol	4.5×10^{-3}[b]	-
R8	Formaldehyde + Et → C_3 + 2H_2O	C_3 pathway from acetone	0.65[b]	-
R9	Acetone + Et → C_3 + Ac + H_2O	C_3 pathway from formaldehyde	5.4×10^{-3}[c]	-
R10	Acetone + Et → C_5 + 2H_2O	C_5 pathway	0.54[b]	-
R11	2Ac + Et → hexatriene + 3H_2O	-	5.0×10^{-4}[c]	-
R12	Et → E + H_2O	Ethanol dehydration	8.0×10^{-3}[a]	157
R13	2Et ⇌ DEE + H_2O	DEE formation	4.4×10^{-4}[b]	103

⇌ Reversible reaction. ⟶ Irreversible reaction. k^0 at 613 K.

Ac – acetaldehyde, MVK – methyl vinyl ketone, Et – ethanol, 2B1OL – 2-buten-1-ol, 3B2OL – 3-buten-2-ol, MEK – methyl ethyl ketone. EtOAc – ethyl acetate, E – ethylene, DEE – diethyl ether.

[a] $m^3 kg_{cata}^{-1}\ s^{-1}$. [b] $(m3)^2 kg_{cata}^{-1}\ mol^{-1}\ s^{-1}$. [c] $(m^3)^3 kg_{cata}^{-1}\ mol^{-2}\ s^{-1}$.

Table S3

Standard ΔH° and ΔG° of reactions involved in proposed mechanisms.

Reactions	ΔH^0, kJ/mol				ΔG^0, kJ/mol			
	298 K	573 K	673 K	773 K	298 K	573 K	673 K	773 K
**One-step process**								
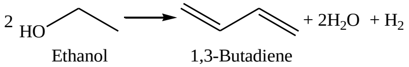	47.8	52.6	53.6	54.3	14.2	-18.8	-31.4	-44.0
**Two-step process**								
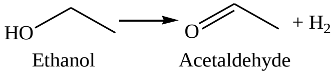	63.9	67.3	68.0	68.5	30.0	-2.6	-14.9	-27.3
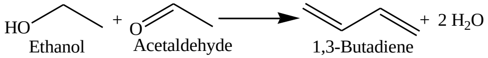	31.6	37.8	39.2	40.2	-1.8	-35.0	-47.8	-60.8
**Egloff and Hulla's mechanism**								
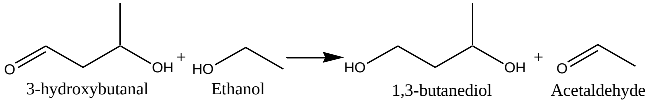	5.7	4.7	4.4	4.1	4.6	4.0	3.9	3.9
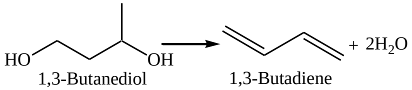	58.8	62.7	63.1	63.2	-15.6	-85.9	-111.9	-137.9
**Ostromislensky mechanism**								
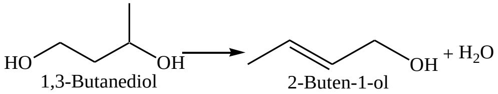	-501.2	-484.9	-478.1	-470.6	-437.7	-384.9	-368.0	-352.1
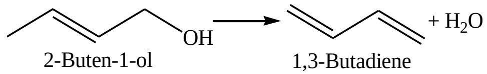	17.6	20.4	20.9	21	-19.4	-54.6	-67.8	-80.9
**Quattlebaum mechanism**								
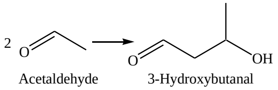	-16.5	-14.8	-14.1	-13.6	4.6	23.5	30.1	36.6
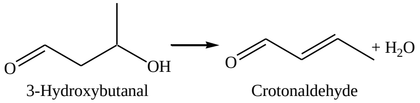	27.3	27.4	27.2	27.1	-4.1	-33.3	-43.8	-34.4
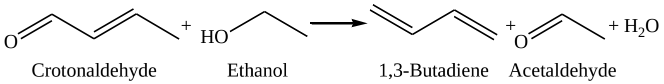	37.3	39.9	40.3	40.3	-6.8	-48.6	-64.1	-79.6
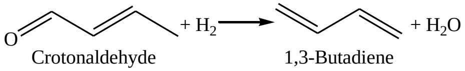	-26.7	-27.4	-27.7	-20.8	-36.9	-45.9	-49.2	-52.3

**Natta and Rigamonti mechanism**								
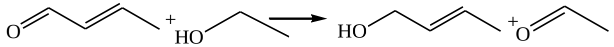 Crotonaldehyde + Ethanol → Crotyl alcohol + Acetaldehyde	19.7	19.5	19.4	19.3	12.6	6.0	3.6	1.3
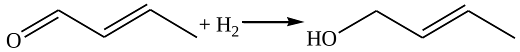 Crotonaldehyde + H_2 → Crotyl alcohol	-44.3	-47.8	-48.6	-49.2	-17.5	8.6	18.5	28.6
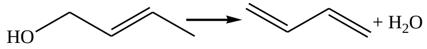 Crotyl alcohol → 1,3-Butadiene + H_2O	17.6	20.4	20.4	20.9	-19.4	-54.6	-67.7	-80.9
**Prins-condensation mechanism**								
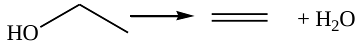 Ethanol → Ethylene + H_2O	45.6	46.9	46.8	46.4	7.7	-27.9	-41.0	-54.0
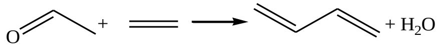 Acetaldehyde + Ethylene → 1,3-Butadiene + H_2O	-14.1	-9.1	-7.5	-6.2	-9.5	-7.0	-6.8	-6.7

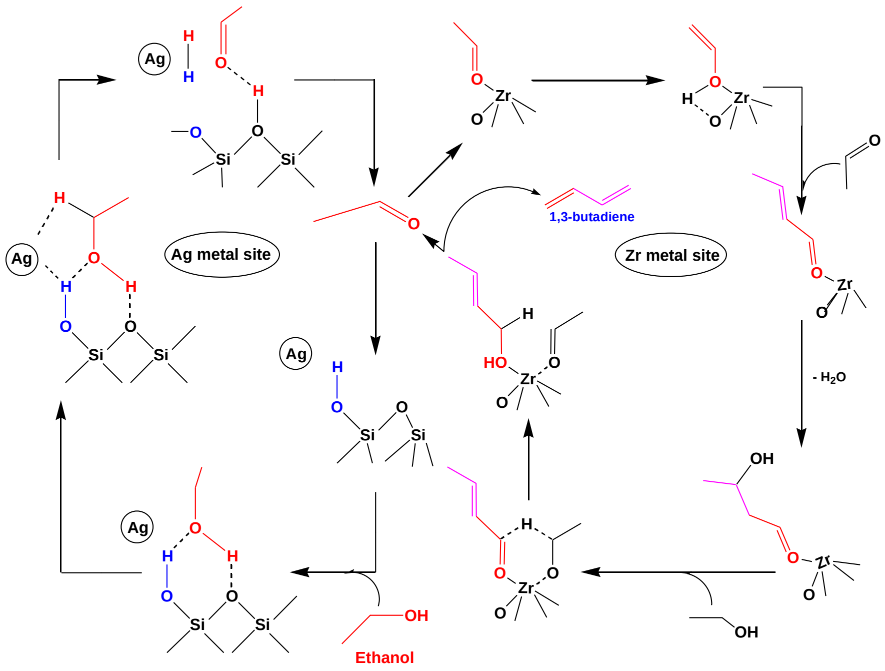

Fig. S1. Dual-cycle mechanism for the conversion of ethanol into 1,3-butadiene.[3]

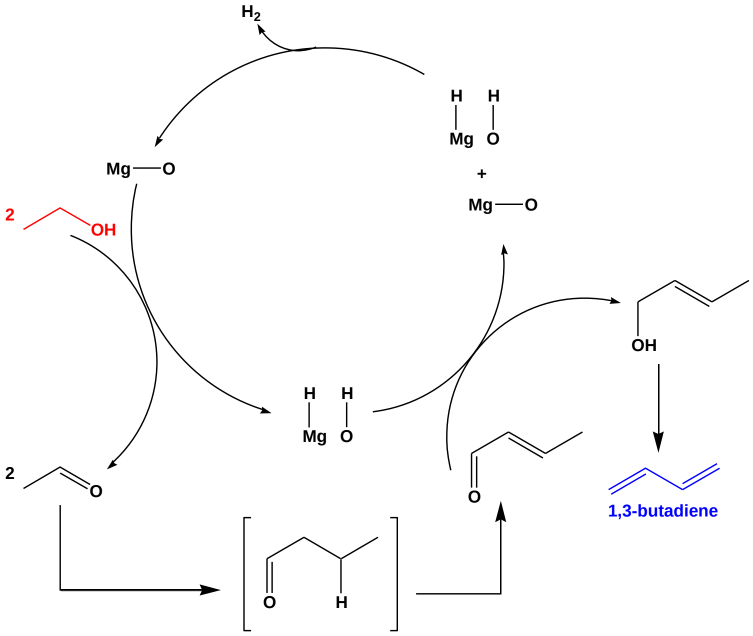

Fig. S2. Reduction of crotonaldehyde to 1,3-butadiene using dissociated hydrogen over MgO catalyst.[4]

References

[1] V. F. Tret'yakov, R. M. Talyshinskii, A. M. Ilolov, A. L. Maksimov, S. N. Khadzhiev, *Pet. Chem.*, 2014, 54, 195–206.

[2] D. Dussol, N. Cadran, N. Laloue, L. Renaudot, J. -M. Schweitzer, *Chem. Eng. J.*, 2020, 391, 123586.

[3] V. L. Sushkevich, I. I. Ivanova, Appl. Catal. B., 2017, 215, 36–49.

[4] A. Miyaji, M. Hiza, Y. Sekiguchi, S. Akiyama, A. Shiga, T. Baba, *J. Jpn. Pet. Inst.*, 2018, 61, 171–81.
